# Accuracy enhancement in the estimation of molecular hydration free energies by implementing the intramolecular hydrogen bond effects

**DOI:** 10.1186/s13321-015-0106-2

**Published:** 2015-11-25

**Authors:** Kee-Choo Chung, Hwangseo Park

**Affiliations:** Department of Bioscience and Biotechnology, Sejong University, 209 Neungdong-ro, Kwangjin-gu, Seoul, 143-747 Republic of Korea

**Keywords:** Hydration free energy, Solvent-contact model, Genetic algorithm, Intramolecular hydrogen bond

## Abstract

**Background:**

The formation of intramolecular hydrogen bonds (IHBs) may induce the remarkable changes in molecular physicochemical properties. Within the framework of the extended solvent-contact model, we investigate the effect of implementing the IHB interactions on the accuracy in estimating the molecular hydration free energies.

**Results:**

The performances of hydration free energy functions including and excluding the IHB parameters are compared using the molecules distributed for SAMPL4 blind prediction challenge and those in Free Solvation Database (FSD). The calculated hydration free energies with IHB effects are found to be in considerably better agreement with the experimental data than those without them. For example, the root mean square error of the estimation decreases from 2.56 to 1.66 and from 1.73 to 1.54 kcal/mol for SAMPL4 and FSD molecules, respectively, due to the extension of atomic parameter space to cope with IHBs.

**Conclusions:**

These improvements are made possible by reducing the overestimation of attractive interactions between water and the solute molecules involving IHBs. The modified hydration free energy function is thus anticipated to be useful for estimating the desolvation cost for various organic molecules.

**Electronic supplementary material:**

The online version of this article (doi:10.1186/s13321-015-0106-2) contains supplementary material, which is available to authorized users.

## Background

Because most biochemical processes take place in aqueous environment, their kinetic and thermodynamic aspects vary with the structural and energetic features of solute-water interactions. Hydration free energy (*ΔG*_*hyd*_) refers to the free energy change for the transfer of a solute molecule in the gas phase to liquid water, and serves as a fundamental quantity to measure the biological activity of organic molecules. For example, *ΔG*_*hyd*_ has been useful for elucidating the strength of protein–ligand association and the efficacy of a drug molecule at the site of action [1–3]. Therefore, the precise estimation of molecular hydration free energy would have the effect of accelerating the pace of drug discovery. The necessity for an accurate computational method for *ΔG*_*hyd*_ prediction has become more urgent because the experimental measurements of *ΔG*_*hyd*_ lagged behind a rapid increase in the number of new organic compounds [4, 5].

Despite the difficulty in describing the complex solute-water interactions, a number of computational methods for *ΔG*_*hyd*_ prediction have been developed based on a variety of theoretical frameworks [6–15]. In 1993, Stouten et al. proposed a simple hydration free energy function constructed within the framework of solvent-contact model [16], which placed an emphasis on the direct relation between *ΔG*_*hyd*_ and solvent-accessible volume around a solute atom [17]. Despite the simplicity in describing the solute-water interactions with the three atomic parameters for only six atom types (C, N, O, N^+^, O^−^, and S), this hydration model was successfully applied to explain the structural properties of small proteins. In the previous studies, we improved the solvent-contact model to make it useful for estimating the *ΔG*_*hyd*_ values of diverse organic molecules by extending the atom types and atomic parameters to cope with a variety of chemical environments [18–20]. A good performance of this extended solvent-contact model was demonstrated in SAMPL4 blind prediction challenge for molecular hydration free energies [21]. In contrast to the successful prediction of the experimental *ΔG*_*hyd*_ values for the majority of organic molecules, the extended solvent-contact model showed a relatively poor performance with respect to the solutes molecules that are capable of establishing the intramolecular hydrogen bonds (IHBs). This imperfection has made it difficult for the hydration free energy function to be useful in practical applications.

Such a defect of the previous extended solvent-contact model is actually not surprising because the formation of IHBs may have a significant influence on the solute-water interactions due to the electron redistribution between the hydrogen-bond donor/acceptor groups. We aim in this study to further improve the solvent-contact model in such a way to precisely estimate the *ΔG*_*hyd*_ values of all the solute molecules including those involving IHBs. For this purpose, the atomic parameter space of the hydration free energy function is extended to reflect the effect of forming IHBs on the strength of solute-water interactions. The presence of a suitably positioned IHB in solute molecules was shown to improve the membrane permeability of a drug molecule in close relation with its solubility in aqueous solution [22]. Therefore, the modified hydration free energy function implementing the IHB effects seems to be useful for estimating the favorable drug-like properties, which further motivates this research.

## Computational methods

Within the framework of the extended solvent-contact model, hydration free energy function for a solute molecule can be written in the following form.

1 $$\varDelta G_{hyd} = \sum\limits_{i}^{atoms} {S_{i} (O_{i}^{max} - \sum\limits_{j \ne i}^{atoms} {V_{j} e^{{\frac{{r_{ij}^{2} }}{{2\sigma^{2} }}}} } )}$$

Here, gaussian-type envelope function with respect to the interatomic distance between solute atoms (*r*_*ij*_) and a constant (*σ*) is employed to define the occupied volume to which the approach of water molecules is restricted. *S*_*i*_, *O*_*i*_^*max*^, and *V*_*j*_ represent the atomic hydration energy per unit volume, the maximum atomic occupancy, and the atomic fragmental volume, respectively. The determination of these three parameters for each atom type is prerequisite for the calculation of *ΔG*_*hyd*_. In this study, we optimized the *S*_*i*_, *O*_*i*_^*max*^, and *V*_*j*_ parameters by means of a standard genetic algorithm using a variety of solute molecules for which the experimental *ΔG*_*hyd*_ values were available. The organic molecules contained in Free Solvation Database (FSD) [23] and those distributed in SAMPL4 blind prediction challenge [24] were used for validating the accuracy of the optimized hydration free energy function.

### Preparation of training and test sets

A total of 643 organic molecules in the latest version of FSD were divided into 439 and 200 molecules to construct the training and test sets, respectively, after excluding the four molecules (ammonia, hydrogen oxide, methylsulfinylmethane, and endosulfan alpha) that included the unique atom types unavailable in the other molecules. With respect to the separation of 639 FSD molecules into a training set and a test set, the similar molecules sharing more than 70 % of atom types were collected into the same structural cluster. For a cluster containing *n* elements, one-third of the molecules were randomly selected as the elements of the test set. If the number of molecules was less than 6 in a structural cluster, we selected only a single molecule as the element of test set to avoid the irrelevant optimization of atomic parameters. Both training and test sets were then confirmed for the inclusion of all the atom types present in FSD. To further investigate the impact of implementing the IHB effects on the accuracy of hydration free energy function, we also used 47 molecules distributed in SAMPL4 blind prediction challenge as the test set along with a training set prepared with 77 organic molecules [21]. All structures of the molecules in training and test sets are presented in Additional file 1.

Whereas the chemical diversity of SAMPL4 dataset is very limited because it includes a small number of molecules distributed as the targets for blind test, FSD contains structurally diverse molecules including more than 40 functional groups. Molecular weight, dipole moment, and experimental hydration free energy range from 16.04 to 498.66 g/mol, from 0 to 7.14 Debye, and from −25.47 to 3.43 kcal/mol, respectively. These wide ranges of structure and physicochemical properties support the reasonableness of selecting FSD to validate the hydration free energy function.

**Fig. 1:**
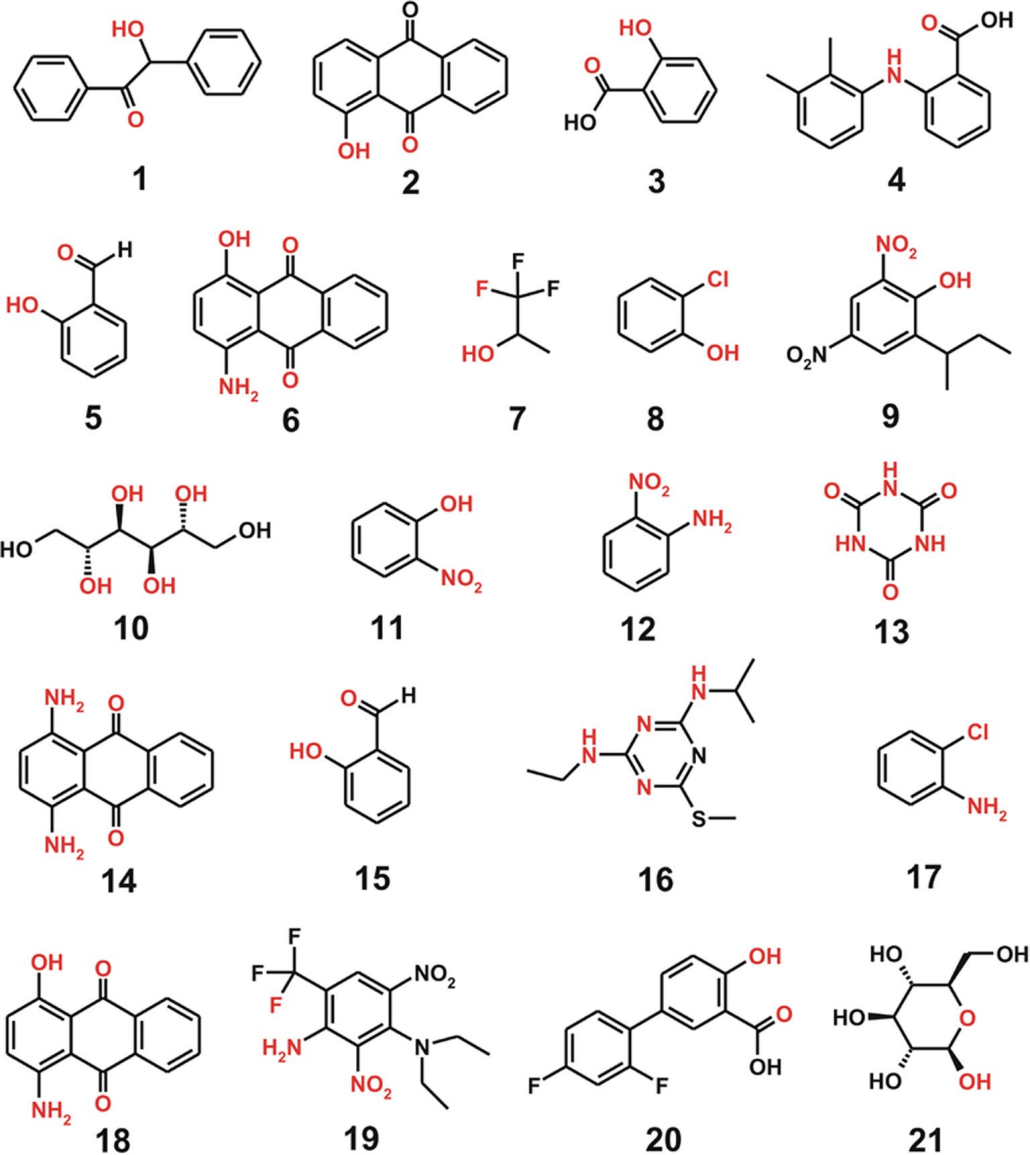
Structures of the solute molecules capable of forming the intramolecular hydrogen bonds. Functional groups involved in the hydrogen bonds are indicated in *red*. **1–3**, **4–6**, **7–13**, and **14–21** belong to training set for SAMPL4, test set of SAMPL4, training set of FSD, and test set of FSD molecules, respectively. 2-Hydroxybenzaldehyde (**5** and **15**) and 1-amino-4-hydroxyanthraquinone (**6** and **18**) are shown in duplicate because they belong to different data sets and their hydration free energies were calculated with different atomic parameters

3-D structures of all the solute molecules required for calculating the *ΔG*_*hyd*_ values were obtained through the quantum chemical geometry optimizations at B3LYP/6-31G* level with polarized continuum model for solvation. The optimized atomic coordinates were then inspected for the presence of IHB, which was defined as the non-bond interaction between polar atom and hydrogen with the interatomic distance shorter than 2.5 Å. Figure 1 shows all the molecules involving IHBs contained in the two training and test sets.

### Definition of atom types

The definition of atom types is critically important in this study because they should reflect all the chemical circumstances each atom in the solute molecules can face. Because the redundant definition of atom types may cause the overfitting during the parametrizations, it is necessary to define the optimal number of atom types to warrant a good performance of the hydration free energy function. In case of SAMPL4 molecules, two additional atom types were required to describe the oxygen and hydrogen atoms involved in IHBs in addition to the existing 34 atom types defined according to the element, hybridization state, chemical bond, and number of substituents. A total of 36 atoms types were therefore needed to describe all the molecules in SAMPL4 data set. The number of atom types increased to 52 to cope with FSD molecules to represent a variety of chemical circumstances in 639 molecules.

### Optimization of atomic parameters

Three key atomic parameters should be determined to calculate the *ΔG*_*hyd*_ values using Eq. (1). Among them, *V*_*i*_ values were fitted separately because they revealed a bad convergent behavior in the simultaneous optimization with *S*_*i*_ and *O*_*i*_^*max*^ parameters. A standard genetic algorithm was employed in the optimization of *V*_*i*_ parameters as detailed in the previous papers [19, 20]. This parameterization could be carried out successfully by minimizing the sum of differences between the van der Waals volume of a solute molecule and the sum of its all atomic *V*_*i*_ parameters. *V*_*i*_ parameters differ from *S*_*i*_ and *O*_*i*_^*max*^ in that they have wide variations among even the same atom types. This exceptional flexibility was assumed in the context that the partial volume of each atom in molecules can vary substantially with the change of the molecular structure irrespective of the atom types.

After the calculation of *V*_*i*_ values for all the atoms in solute molecules, *S*_*i*_ and *O*_*i*_^*max*^ parameters were optimized simultaneously based on the genetic algorithm using the 3-D structures and the experimental *ΔG*_*hyd*_ values of the molecules in the training sets. This began with the construction of a generation consisting of 100 vectors whose elements were *S*_*i*_ and *O*_*i*_^*max*^ parameters for all possible atom types. In the second step, the half of 100 vectors was made empty with a bias toward preserving the best fit with the minimum error. These empty vectors were then filled with the new elements prepared from those of top 50. We generated the 50 new vectors in two steps. First, all *S*_*i*_ and *O*_*i*_^*max*^ values in the filled vectors were altered with probability 0.01 to make the transiently new vectors. The elements of these temporary vectors were then exchanged by cross breeding with probability 0.6 to replace some *S*_*i*_ and *O*_*i*_^*max*^ values with those in another vector. The 50 new vectors constructed in this way were finally scored together with the previous top 50 to select the new top 50. This procedure was iterated until the convergence criterion was satisfied. To score the vectors containing *S*_*i*_ and *O*_*i*_^*max*^ parameters as the elements, we used the error hypersurface (*F*_*s*_) given by summing the discrepancies between the experimental (*ΔG*_*exp*_^*i*^) and calculated molecular hydration free energies (*ΔG*_*calc*_^*i*^). This fitness function can be expressed as follows.

2 $$F_{s} = \sum\limits_{i}^{molecules} {\left| {\varDelta G_{\exp }^{i} - \varDelta G_{calc}^{i} } \right|}$$

During the operation of genetic algorithm, the atomic parameters exhibited convergent behavior after about 2000 iterations.

## Results and discussion

The hydration free energy function was optimized and validated using the two data sets. One contains 639 FSD molecules that were divided into 439 and 200 to constitute the training and test sets, respectively, and the other consists of 77 reference molecules (training set) and 47 SAMPL4 molecules (test set). Prior to the optimization of atomic parameters, we defined a total of 52 and 36 atom types to represent a variety of chemical circumstances in FSD and SAMPL4 molecules, respectively. Some abnormal atom types were required for coping with FSD molecules such as hexavalent sulfur (S.12) and pentavalent phosphorus (P.10) atoms. O**–**H type IHBs were found both in FSD and in SAMPL4 molecules while F**–**H and Cl**–**H forms were present in the former only. These IHBs were identified by the conformational searches for the presence of non-bond interactions between hydrogen and polar heavy atoms with the interatomic distance shorter than 2.5 Å.

Table 1 lists the optimized *O*_*i*_^*max*^ and *S*_*i*_ values for 52 and 36 atom types defined to represent all the molecules in FSD and SAMPL4 data sets, respectively. Despite the large structural and populational differences in the constituent molecules, the *O*_*i*_^*max*^ and *S*_*i*_ values optimized with 439 FSD molecules compare reasonably well with those obtained using 77 molecules to represent 47 SAMPL4 molecules. The squared linear correlation coefficients (*R*^*2*^) to compare the parametrizations with the two training sets amount to 0.79 and 0.83 for *O*_*i*_^*max*^ and *S*_*i*_ values, respectively. The atomic *V*_*i*_ parameters are omitted in Table 1 because they were allowed to vary in accordance with the position in molecules even in the case of the same atom types. In the strict sense, each atom in all the molecules may have its own unique *V*_*i*_ value.

**Table 1:** The optimized maximum atomic occupancy (*O*_*i*_^*max*^) and atomic solvation parameters (*S*_*i*_) for all the atom types defined for FSD and SAMPL4 molecules

Atom type	Description	$O_{i}^{max}$ (Å^3^)		$S_{i}$ (kcal/mol Å^3^)	
		FSD	SAMPL4	FSD	SAMPL4
C.3_1	sp^3^ carbon with 1 substituent	396.8	350.8	0.429	1.619
C.3_2	sp^3^ carbon with 2 substituents	372.4	368.3	0.524	0.143
C.3_3	sp^3^ carbon with 3 substituents	361.9	382.5	−0.429	0.095
C.3_4	sp^3^ carbon with 4 substituents	379.4	377.0	1.222	0.794
C.2_1	sp^2^ carbon with 1 substituent	360.3	339.5	2.048	0.905
C.2_2	sp^2^ carbon with 2 substituents	365.1	354.8	−0.905	0.873
C.2_3	sp^2^ carbon with 3 substituents	391.3	353.8	−1.222	−0.540
C.1_1	sp carbon with 1 substituent	377.0	NA	−0.905	NA
C.1_2	sp carbon with 2 substituents	351.6	NA	0.143	NA
C.ar_2	Aromatic carbon with 2 substituents	392.9	381.1	−1.000	−0.889
C.ar_3	Aromatic carbon with 3 substituents	375.4	353.2	−0.048	0.524
C.CO_1	Carbonyl carbon with 1 substituent	337.1	354.0	−3.968	−2.619
C.CO_2	Carbonyl carbon with 2 substituents	393.3	369.0	−6.444	−1.746
N.1_1	sp nitrogen with 1 substituent	404.0	NA	−10.079	NA
N.2_2	sp^2^ nitrogen with 2 substituents	424.4	NA	−11.556	NA
N.3_1	sp^3^ nitrogen with 1 substituent	351.6	384.9	−9.333	−10.318
N.3_2	sp^3^ nitrogen with 2 substituents	437.6	364.4	−10.238	−10.333
N.3_3	sp^3^ nitrogen with 3 substituents	454.6	393.7	−14.921	−12.302
N.ar	Aromatic nitrogen	357.8	352.4	−8.222	−11.349
N.pl_1	Planar nitrogen with 1 substituent	396.8	358.9	−10.159	−12.460
N.pl_2	Planar nitrogen with 2 substituents	330.0	367.5	−10.873	−11.667
N.pl_3	Planar nitrogen with 3 substituents	358.7	408.9	−8.444	−11.905
N.am_1	Amide nitrogen with 1 substituent	398.9	NA	−8.429	NA
N.am_2	Amide nitrogen with 2 substituents	391.1	NA	−9.603	NA
N.am_3	Amide nitrogen with 3 substituents	399.2	NA	−3.635	NA
N.no2	Nitrogen in nitro group	357.9	372.2	−4.444	−4.921
O.3_1	sp^3^ oxygen with 1 substituent	330.8	366.2	−13.556	−11.619
O.3_2	sp^3^ oxygen with 2 substituents	304.4	311.4	−5.714	−5.873
O.pl_1	Planar oxygen with 1 substituent	NA	316.2	NA	−10.619
O.pl_2	Planar oxygen with 2 substituents	NA	346.8	NA	−6.825
O.es_1	sp^3^ oxygen in carboxylic acids	309.5	327.8	−6.508	−8.413
O.es_2	sp^3^ oxygen in esters	319.8	333.3	1.778	−2.603
O.2	sp^2^ oxygen	302.4	347.6	−7.619	−9.683
O.no2	Oxygen in nitro group	342.1	338.9	−0.476	0.825
O.intra	Oxygen involved in intramolecular hydrogen bond	323.0	309.0	−1.270	−3.810
S.12	Sulfur with 12 valence electrons	410.3	NA	−3.810	NA
S.3_1	sp^3^ sulfur with 1 substituent	429.4	NA	−0.762	NA
S.3_2	sp^3^ sulfur with 2 substituents	402.4	NA	−6.857	NA
S.2	sp^2^ sulfur	428.6	NA	1.556	NA
S.pl	Planar sulfur	409.5	NA	−0.190	NA
F	Fluorine	284.1	NA	−3.714	NA
F.intra	Fluorine involved in intramolecular hydrogen bond	277.9	NA	1.365	NA
Cl	Chlorine	452.4	408.7	−0.794	−3.016
Cl.intra	Chlorine involved in intramolecular hydrogen bond	458.1	NA	−0.317	NA
Br	Bromine	500.8	NA	−1.778	NA
I	Iodine	549.2	NA	−1.556	NA
P.10	Phosphorus with 10 valence electrons	404.4	NA	−4.095	NA
H.C	Hydrogen bonded to carbon	201.6	182.2	0.111	−0.444
H.N3	Hydrogen bonded to sp^3^ nitrogen	254.4	212.7	−5.556	−2.540
H.Np	Hydrogen bonded to planar nitrogen	207.9	223.8	−1.746	−1.159
H.O3	Hydrogen bonded to sp^3^ oxygen	236.7	204.8	−7.159	−9.286
H.Op	Hydrogen bonded to planar oxygen	NA	237.3	NA	−10.889
H.Oa	Hydrogen bonded to carboxylic acid group	230.2	202.4	−3.444	−6.190
H.S	Hydrogen bonded to sulfur	228.9	NA	−5.397	NA
H.intra	Hydrogen involved in intramolecular hydrogen bond	222.2	210.0	−3.190	−4.222

Despite the structural diversity of the molecules in the training sets, the optimized atomic parameters have a tendency consistent with general atomic properties. We note in this regard that the *O*_*i*_^*max*^ values appear to get larger with the increase in atomic radius from hydrogen to the second- and third-period elements. Oxygen and fluorine atoms have the lower *O*_*i*_^*max*^ values than carbon and nitrogen, which is consistent with the smaller atomic radii of the former than the latter. Thus, we can obtain the physically reasonable *O*_*i*_^*max*^ values through the definitions of 52 and 36 atoms types for FSD and SAMPL4 molecules, respectively.

In contrast to the relative similarities among the *O*_*i*_^*max*^ values for varying atom types, the *S*_*i*_ parameters appear to undergo a large change with the variation of atom types even in the case of the same element. Nonetheless, the optimized *S*_*i*_ values also exhibit a trend that can be elucidated with the electronic structures of individual atoms. For example, various carbon atoms have positive or very low negative *S*_*i*_ values in both parametrizations with FSD and SAMPL4 molecules, which indicates their insignificant interactions with water. This is consistent with the low solubility of hydrocarbons in water. However, the decrease of the average *S*_*i*_ values in the order of *sp*^*3*^, *sp*^*2*^, and *sp* carbons indicates that the interaction of a solute carbon atom with water becomes more favorable due to the increase of the s-character in the hybridization state of atomic orbitals. Such a dependence of *S*_*i*_ on the extent of s-character may be elucidated in the context that the increased s-character in the hybrid atomic orbitals has the effect of increasing the electronegativity, which would culminate in facilitating the intermolecular dipole–dipole interactions with water. Besides the increased electronegativity, the decrease in the number of substituents on the carbon with high s-character would also have the effect of lowering the *S*_*i*_ value because water molecules can approach the central carbon readily along a line perpendicular to the molecular plane. Due to the combined effects of the increased polarity and the increased water accessibility, both atom types of carbonyl carbons (C.CO_1 and C.CO_2) have very negative *S*_*i*_ values. This is physically acceptable in terms of the high solubility of carbonyl compounds in water.

Consistent with the critical roles of nitrogen and oxygen atoms in the stabilization of organic molecules in water, their optimized *S*_*i*_ values are highly negative for most atom types. This may be invoked to explain the long-range attractive electrostatic interactions with bulk water and to the capability to form the local hydrogen bonds with water molecules, both of which contribute to making the solute-water interactions thermodynamically favorable. However, the *S*_*i*_ values of oxygens appear to become less negative in the presence of IHBs in solute molecules. The optimized *S*_*i*_ parameters of the oxygen atoms involved in IHBs (O.intra) amount to −1.270 and −3.810 in the parametrizations for FSD and SAMPL molecules, respectively, as compared to the corresponding average *S*_*i*_ values of −5.349 and −6.851 for the rest of oxygens. This can be related with the partial loss of electron density on the oxygen due to the electron transfer from its non-bond orbital to the antibonding σ* orbital of hydrogen-bond donor group, which is characteristic of a normal hydrogen bond.

The *S*_*i*_ values of the hydrogen atoms bonded to heteroatoms are much more negative than those of hydrocarbons (Table 1). This is consistent with the accumulation of positive charges due to the electron withdrawal by the neighboring heteroatoms that are more electronegative than carbon. In accordance with the increase of *S*_*i*_ value for O.intra, however, the H.intra atoms also reveal the less negative *S*_*i*_ values than the other hydrogens adjacent to the heteroatoms. For instance, the *S*_*i*_ parameter of H.intra converges to −3.190 and −4.222 in the optimization with FSD and SAMPL4 molecules, respectively, in comparison to the corresponding average values of −4.660 and −6.013 for the other hydrogens attached to the electronegative heteroatoms. This can be elucidated also in the context of the electron transfer from the hydrogen-bond acceptor atom and the resulting partial neutralization of the positive charges on H.intra atoms.

**Fig. 2:**
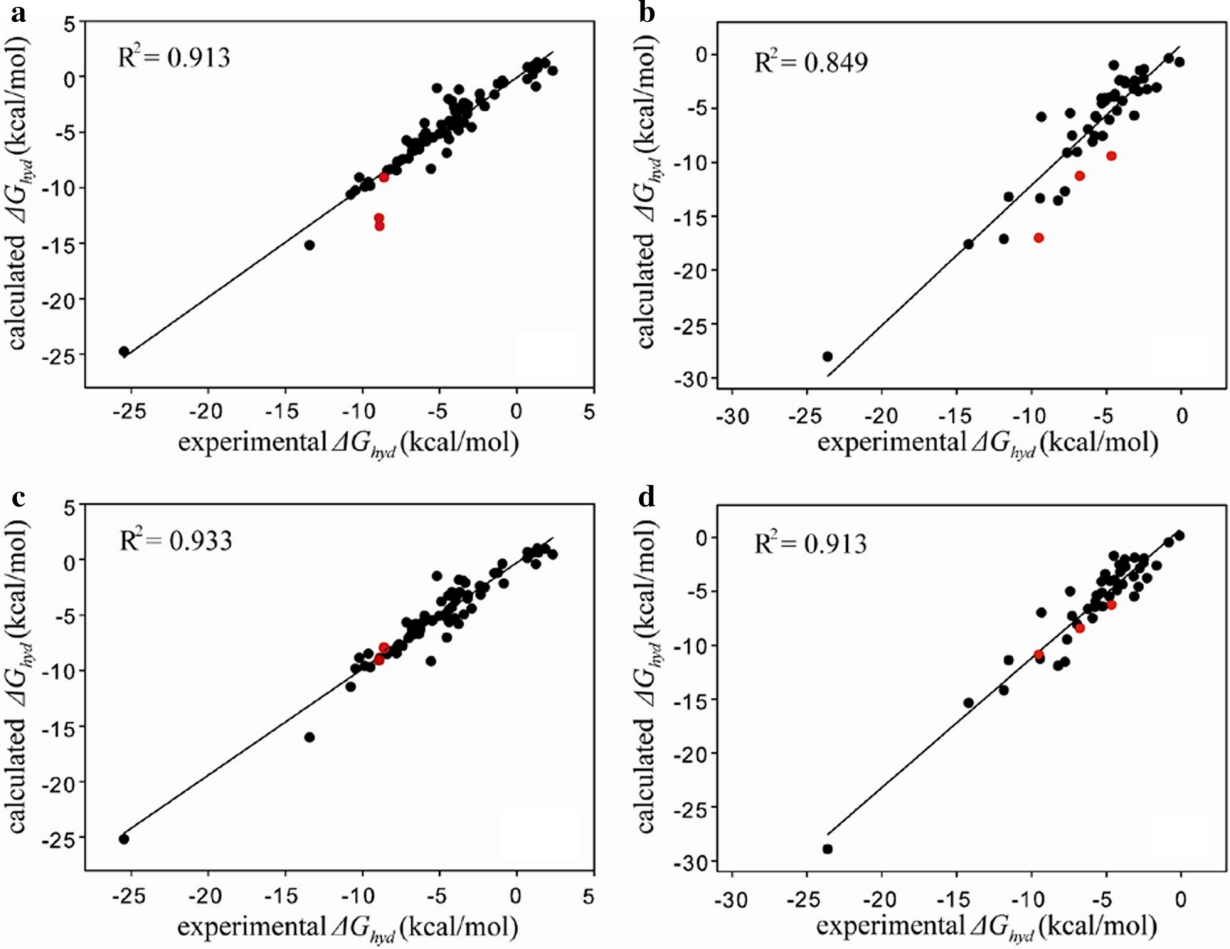
Linear correlation diagrams for the experimental versus calculated hydration free energies for **a** training set of 77 molecules without IHB parameters, **b** test set of 47 SAMPL4 molecules without IHB parameters, **c** training set of 77 molecules with IHB parameters, and **d** test set of 47 SAMPL4 molecules with IHB parameters. Indicated in *red circles* are the solute molecules involving IHBs

Figure 2 shows the linear correlation diagrams between the experimental hydration free energies and those calculated with the optimized hydration free energy function with respect to the training and the test set comprising 77 reference molecules and 47 SAMPL4 molecules, respectively. To examine the effect of parameterizing IHBs in solute molecules on the accuracy of hydration free energy function, we compare the results of *ΔG*_*hyd*_ prediction with the atomic parameters for IHBs to those without them. With respect to the test set consisting of 47 SAMPL4 molecules, we obtain the *R*^*2*^ value of 0.849 in the absence of IHB parameters (Fig. 2b). However, the *R*^*2*^ value of the fitting for the test set increases to 0.913 due to the reflection of IHB effects in the parametrization (Fig. 2d). When the positions of the solute molecules involving IHB in the fitting (red circles in Fig. 2) are compared, it follows immediately that the major contribution to the enhancement of the correlation comes from the better estimation of their *ΔG*_*hyd*_ values. It is also noteworthy that the extension of atomic parameter space to cope with IHBs leads to the decrease in the difference between the *R*^*2*^ values of the training and test sets from 0.064 to 0.020, which indicates the reduced possibility of overtraining during the operation of genetic algorithm. Furthermore, the root mean square error (RMSE) for estimating the *ΔG*_*hyd*_ values of SAMPL4 molecules appears to decrease substantially from 2.56 to 1.66 kcal/mol due to the additional parameterization for IHBs. These results exemplify the necessity of separate atomic parameters for IHBs in solute molecules to improve the accuracy in estimating the molecular hydration free energies.

**Fig. 3:**
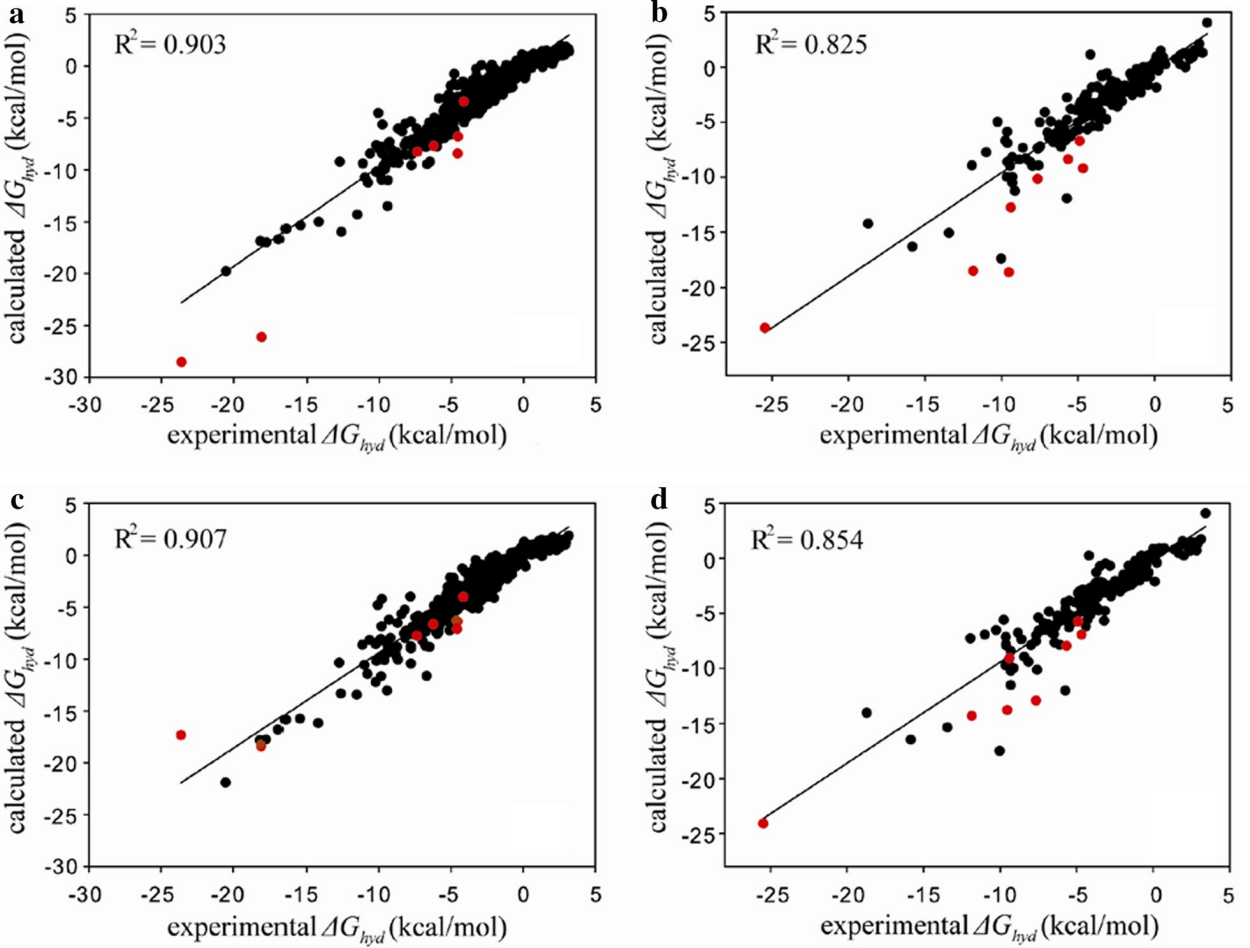
Linear correlation diagrams for the experimental versus calculated hydration free energies for **a** training set of 439 FSD molecules without IHB parameters, **b** test set of 200 FSD molecules without IHB parameters, **c** training set of 439 FSD molecules with IHB parameters, and **d** test set of 200 FSD molecules with IHB parameters. Indicated in *red circles* are the solute molecules involving IHBs

The importance of implementing the IHB effects in estimating the *ΔG*_*hyd*_ values is further demonstrated in the validation results for FSD molecules. Figure 3 illustrates the correlations between the *ΔG*_*hyd*_ values of FSD molecules measured from experiments and those calculated with Eq. (1) and the optimized atomic parameters. It is a common feature in the fittings with SAMPL4 and FSD data sets that the improvement of *R*^*2*^ value due to the augmentation of the atomic parameters is even more significant in the test set than in the training set. We obtain the *R*^*2*^ value of 0.825 without the IHB parameters for the test set comprising 200 molecules (Fig. 3b), as compared to 0.903 for the training set of 439 molecules. This large difference in the *R*^*2*^ values implies that the atomic parameters should be over-trained in the absence of the atom types for IHB. The *R*^*2*^ value of the test set appears to increase to 0.854 in the fitting for the *ΔG*_*hyd*_ results obtained under consideration of IHB effects (Fig. 3d). This significant predictability enhancement can be attributed in a large part to the better prediction of the *ΔG*_*hyd*_ values of the solute molecules with IHBs, which can be inferred from their positional shifts in the linear correlation diagrams (red circles in Fig. 3). Due to the additional parameterization for IHBs, RMSE for the predicted *ΔG*_*hyd*_ values of test set molecules amounts to only 1.54 kcal/mol, as compared to 1.73 kcal/mol in the hydration model excluding the IHB effects. The significant enhancements in *R*^*2*^ and RMSE values confirm the necessity for extending the atomic parameter space to cope with IHBs for the better estimation of molecular hydration free energies.

As can be inferred from the decrease of the *R*^*2*^ value from 0.913 (Fig. 2d) to 0.854 (Fig. 3d), our extended solvent-contact model exhibits a worse performance for FSD molecules than for SAMPL4 ones in terms of the correlation with the experimental data. This may be attributed to the requirement of much more atom types for FSD than for SAMPL4 molecules because chemical environments are more diverse in the former than in the latter. Furthermore, we find that some atom types for sulfur, *sp* carbon, and *sp*^*2*^ nitrogen atoms are rarely observed in FSD data set, which makes it difficult for the corresponding atomic parameters to be fully optimized due to the insufficient number of representatives in the training set.

**Fig. 4:**
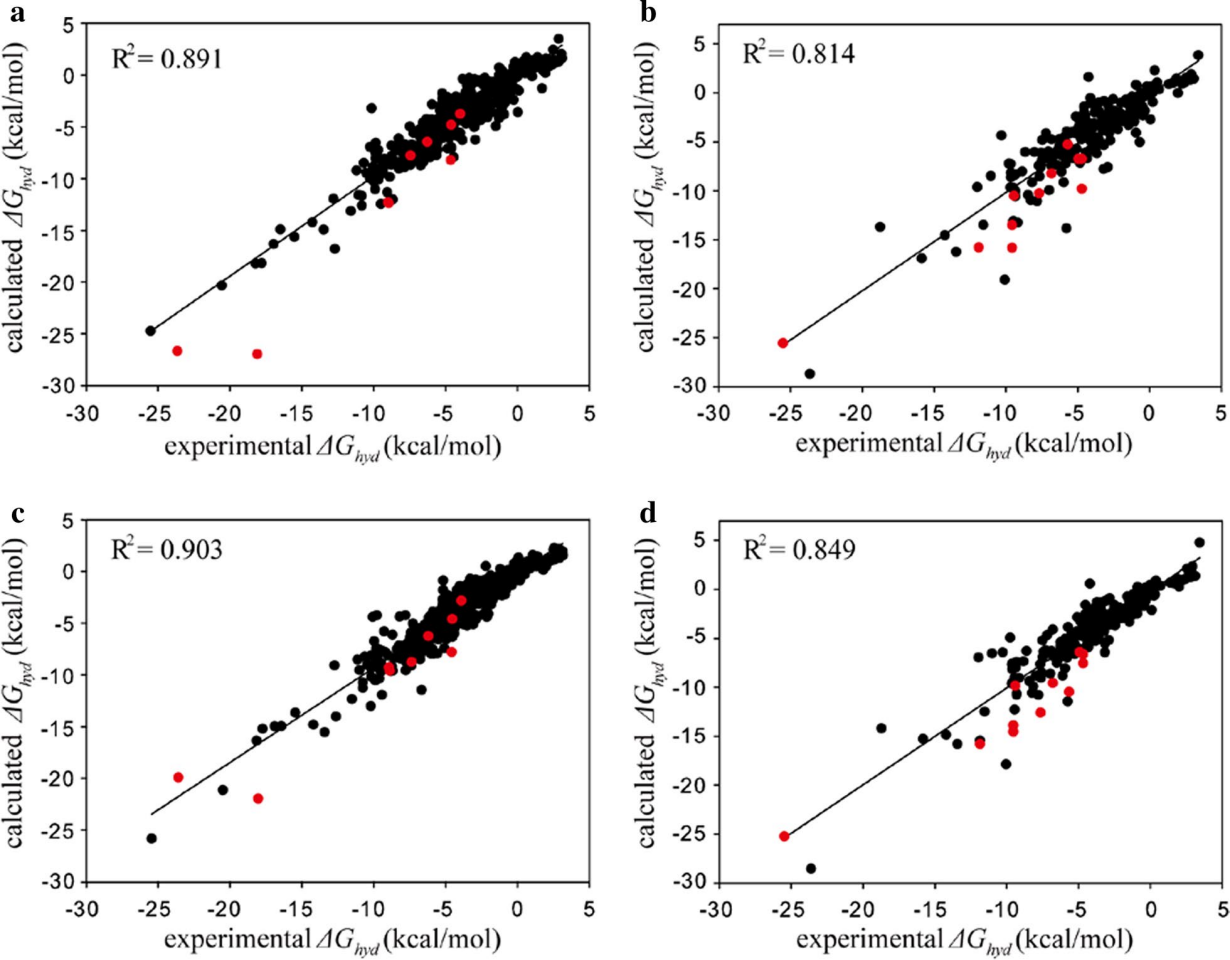
Linear correlation diagrams for the experimental versus calculated hydration free energies for **a** training set of a total of 516 molecules without IHB parameters, **b** test set of 247 SAMPL4 and FSD molecules without IHB parameters, **c** training set of 516 molecules with IHB parameters, and **d** test set of 247 SAMPL4 and FSD molecules with IHB parameters. Indicated in *red circles* are the solute molecules involving IHBs

The performance of hydration free energy function was further evaluated using the new training and test sets constructed by merging those for SAMPL4 and FSD datasets, the results of which are summarized in Fig. 4. The *R*^*2*^ value of 0.814 is obtained for the test set comprising 47 SAMPL4 plus 200 FSD molecules in the absence of the IHB parameters (Fig. 4b), which is even smaller than that (0.891) for the training set comprising a total of 516 (77 plus 439) molecules (Fig. 4a). Judging from such a large difference in the *R*^*2*^ values, the atomic parameters seem to be over-trained in the absence of the IHB parameters. The *R*^*2*^ value of the test set increases to 0.849 in the fitting for the experimental and computational *ΔG*_*hyd*_ values if the IHB effects are reflected in the parametrizations (Fig. 4d). Furthermore, the augmentation of the IHB atomic parameters leads to the decrease of RMSE for *ΔG*_*hyd*_ predictions of the new test set molecules from 1.94 to 1.68 kcal/mol. The validation results obtained with the merged dataset are thus consistent with those for SAMPL4 and FSD datasets in the context that *R*^*2*^ and RMSE values increase and decrease, respectively, due to the implementation of IHB effects. This consistency confirms that the extension of atomic parameter space is necessary to enhance the accuracy in estimating the *ΔG*_*hyd*_ values of the solute molecules involving IHBs.

Compared in Table 2 are the experimental and computational *ΔG*_*hyd*_ values of the solute molecules (**1**–**21** shown in Fig. 1) that involve IHBs. A high discrepancy between experimental and computational results is observed for most IHB molecules that belong to the test sets (**4**–**6** and **14**–**21**) if the IHB effects are neglected. The average unsigned error (AUE) of the calculated *ΔG*_*hyd*_ values for these solute molecules amounts to 4.12 kcal/mol, which is much higher than that for all the molecules included in the two test sets (1.28 kcal/mol). The most inaccurate result is obtained with the deviation of 9.11 kcal/mol for **18** in which multiple IHBs are established.

**Table 2:** Experimental (*ΔG*_*hyd,exp*_) and calculated (*ΔG*_*hyd,calc*_) hydration free energies (in kcal/mol) of 21 molecules capable of establishing the intramolecular hydrogen bonds

Compound	*ΔG*_*hyd,exp*_	No IHB parameter		IHB parameters included	
		*ΔG*_*hyd,calc*_	Error	*ΔG*_*hyd,calc*_	Error
**1**	−8.62	−9.05	0.43	−7.97	0.65
**2**	−8.90	−13.46	4.56	−8.86	0.04
**3**	−8.95	−12.74	3.79	−9.08	0.13
**4**	−6.78	−11.28	4.50	−8.43	1.65
**5**	−4.68	−9.41	4.73	−6.25	1.57
**6**	−9.53	−17.03	7.50	−10.87	1.34
**7**	−4.16	−3.41	0.75	−4.01	0.15
**8**	−4.55	−6.80	2.25	−6.38	1.83
**9**	−6.23	−7.71	1.48	−6.65	0.42
**10**	−23.62	−28.50	4.88	−17.32	6.30
**11**	−4.58	−8.43	3.85	−7.12	2.54
**12**	−7.37	−8.25	0.88	−7.76	0.39
**13**	−18.06	−26.12	8.06	−18.40	0.34
**14**	−11.85	−18.50	6.65	−14.30	2.45
**15**	−4.68	−9.18	4.50	−6.92	2.24
**16**	−7.65	−10.14	2.49	−12.90	5.25
**17**	−4.91	−6.71	1.80	−5.73	0.82
**18**	−9.53	−18.64	9.11	−13.79	4.26
**19**	−5.66	−8.36	2.70	−7.95	2.29
**20**	−9.40	−12.74	3.34	−9.06	0.34
**21**	−25.47	−23.69	1.78	−24.08	1.39

**1–3**, **4–6**, **7–13**, and **14–21** belong to training set for SAMPL4, test set of SAMPL4, training set of FSD, and test set of FSD molecules, respectively

It is a common feature in the *ΔG*_*hyd*_ values of most IHB molecules calculated without the IHB parameters that they are underestimated substantially when compared to the corresponding experimental results. This indicates that the large errors in the calculated *ΔG*_*hyd*_ values of IHB molecules stem from the overestimation of attractive solute-water interactions. However, the implementation of IHB parameters leads to a dramatic decrease in the discrepancies between the experimental and calculated *ΔG*_*hyd*_ values of the solute molecules capable of forming IHBs. For example, the AUE value for **4**–**6** and **14**–**21** decreases to only 2.01 kcal/mol due to the additional parameterizations for IHB, which is relatively similar to that for all the solute molecules in the two test sets (1.09 kcal/mol). Thus, the accuracy enhancement in the present extended-solvent contact model can be attributed to the alleviation of the overestimation of the attractive solute-water interactions.

Related with the substantial contribution of IHBs to molecular hydration free energy, it needs to be noted that the experimental *ΔG*_*hyd*_ value increases from −11.85 in **14** to −9.53 kcal/mol in **18** in response to the replacement of –NH_2_ with –OH moiety. This is quite unexpected because the *ΔG*_*hyd*_ value of aniline (−5.49 kcal/mol) is higher than that of phenol (−6.61 kcal/mol). In this regard, we obtain a slightly higher *ΔG*_*hyd*_ value for **14** than for **18** in the absence of IHB parameters, which is more consistent with the experimental results for aniline and phenol than those for **14** and **18**. On the other hand, the *ΔG*_*hyd*_ value of **14** becomes more negative than that of **18** if they are calculated with the hydration free energy function implementing the IHB parameters. It can thus be argued that the relative strength of solute-water interactions for IHB molecules may be predicted incorrectly in the absence of IHB parameters. The governing role of IHB in the hydration behaviors of solute molecules was also observed in the experimental measurements of dielectric relaxation [25].

The higher *ΔG*_*hyd*_ value of **18** than **14** can be understood in terms of the difference in the strength of IHB. Because phenolic group is more acidic than anilinic one, the former should form the stronger hydrogen bond with the vicinal carbonyl oxygen than the latter. Therefore, a substantial amount of electron density seems to be transferred from the non-bonding orbital of the carbonyl oxygen to the anti-bonding σ* molecular orbital of phenolic O–H bond, which has the effect of lowering the polarities of both chemical moieties involved in IHB. This culminates in the weakening of long-range attractive electrostatic interactions with bulk solvent as well as in reducing the possibility of forming local hydrogen bonds with water molecules. In this viewpoint, the problem of overestimating the attractive solute-water interactions seems to be inevitable unless the IHB effects are taken into account in the hydration free energy function.

It should be noted that some atom types such as S.pl and F.intra (Table 1) are rare in the training set. The low occurrence of certain atom types in the training set may affect the accuracy of hydration free energy function. For example, the differences between the experimental and calculated hydration free energies of **16** and **19** amount to 69 and 40 %, respectively (Table 2). These large deviations can be attributed to the incomplete optimization of atomic parameters due to the low occurrence of S.pl and F.intra in the training set.

Actually, the accuracy in estimating the *ΔG*_*hyd*_ values can be enhanced by increasing the number of atom types in such a way to cope with all the solute atoms in different chemical environments. For example, it would be desirable to distinguish the carbonyl carbons from the normal sp^2^ carbons to reflect the significant positive atomic charge developed due to the adjacent carbonyl oxygen. Some additional atoms types for nitrogen and oxygen seem to be required as well for drug-like molecules because they include a variety of heterocyclic moieties. However, the subdivision of atom types may have a negative effect on the accuracy when the experimental data for training are insufficient for optimizing the parameters associated with the newly created atom types. For example, the low occurrences of S.pl and F.intra atoms in the training set lead to a large deviation of the calculated *ΔG*_*hyd*_ values of **16** and **19** from the experimental ones (Table 2) due to the incomplete optimization of atomic parameters. To maximize the accuracy in *ΔG*_*hyd*_ predictions, therefore, the extension of atom types should be limited to the cases for which the atomic parameters can be fully optimized with the corresponding experimental data.

The merit of the present extended solvent-contact model lies in the capability to elucidate the unusual possibility that the substitution of a more polar moiety than the existing one may render the solute molecule more hydrophobic due to the formation of IHBs. In this regard, some peptidomimetic molecules proved to become more hydrophobic with the substitution of two polar groups to establish an IHB, which led to the enhancement of membrane permeability without impairing the other drug-like properties [22]. The hydration free energy function implementing the IHB parameters is therefore anticipated to be useful for screening drug candidates with good membrane permeability.

However, the present hydration model seems to be a little imperfect as a useful *ΔG*_*hyd*_ estimator because the errors for some IHB molecules amount to more than 4 kcal/mol (Table 2). With respect to this large deviation, we note that the experimental *ΔG*_*hyd*_ data are available only for a small number of IHB molecules in publicly accessible chemical databases. It is therefore difficult to fully optimize the IHB parameters due to the rarity of reference data. We expect that the performance of our extended solvent-contact model will be further enhanced considerably in the future in the presence of abundant experimental *ΔG*_*hyd*_ data for IHB molecules.

Despite the difficulty in collecting the reference data, the hydration free energy function appears to be improved to a significant extent due to the implementation of atomic IHB parameters as illustrated in Figs. 2 and 3. This improvement is made possible because the risk of over-fitting due to the increased atomic parameters can be surmounted effectively by reducing the overestimation of attractive interactions between water and IHB molecules. The RMSE values of 1.66 and 1.54 kcal/mol associated with *ΔG*_*hyd*_ prediction for SAMPL4 and FSD molecules, respectively, seem to be insignificant because the experimental *ΔG*_*hyd*_ data cover a wide range of ~ 30 kcal/mol. The accuracy of the present extended solvent-contact model is comparable in terms of *R*^*2*^ value to those of some high-level quantum chemical calculations and statistical simulations with all-atom models [26–29]. The characteristic feature that discriminates our hydration model from the others lies in that one can compute the *ΔG*_*hyd*_ values in a straightforward way from the potential energy function without significant computational burden.

Although it is shown in this study that *ΔG*_*hyd*_ values can be estimated effectively with the extended solvent-contact model implementing the IHB effects, there remains the possibility of further improvement. For example, the entropic contribution to *ΔG*_*hyd*_ needs to be calculated separately because the hydration free energy function in Eq. (1) lacks the entropic term. Although the determination of hydration entropy (*ΔS*_*hyd*_) had been considered a very difficult task, it was reported recently that *ΔS*_*hyd*_ could be estimated with reasonable accuracy by means of combining free energy perturbation (FEP) method and scaled particle theory (SPT) to calculate the electrostatic and hydrophobic contributions of solute-water interactions separately [30]. Therefore, the combination of *ΔH*_*hyd*_ and *ΔS*_*hyd*_ values calculated respectively with our extended solvent-contact model and the hybrid SPT/FEP approach seems to serve as a useful method for estimating the *ΔG*_*hyd*_ values of small molecules.

## Conclusions

The formation of IHBs in solute molecules may lead to the weakening of solute-water interactions due to the charge transfer between the hydrogen-bond acceptor/donor groups. This would have the effect of reducing the polarity of solute molecules, and cause the unexpected increase in the *ΔG*_*hyd*_ values. In this study, we examined the effect of implementing the IHB interactions on the accuracy of the extended solvent-contact model for *ΔG*_*hyd*_ prediction using SAMPL4 and FSD molecules. As a consequence of augmenting the atomic parameters for IHBs, the calculated *ΔG*_*hyd*_ values became in better agreement with experimental data. For example, the *R*^*2*^ values between the experimental and calculated *ΔG*_*hyd*_ values increased from 0.849 to 0.913 and from 0.825 to 0.854 for SAMPL4 and FSD molecules, respectively, due to the extension of atomic parameters to cope with IHBs. Furthermore, the RMSE values of the estimation decreased from 2.56 to 1.66 kcal/mol and from 1.73 to 1.54 kcal/mol for SAMPL4 and FSD molecules, respectively, in the presence of the atomic IHB parameters. The comparisons of the calculated *ΔG*_*hyd*_ values indicated that such a significant accuracy enhancement stemmed from the reduction of the overestimation for the attractive electrostatic interactions between water and IHB molecules. This was in turn made possible by properly describing the electron redistribution between IHB acceptor and donor groups, which has the effect of weakening their polarities. Thus, the results in this study exemplified the necessity for the augmentation of atomic parameters according to the specific chemical environments to improve the accuracy of the hydration free energy function.

## Additional file

10.1186/s13321-015-0106-2 Contains chemical structures, experimental and calculated solvation free energies of 763 molecules used in this study.
