# Supplementary material for: Accuracy enhancement in the estimation of molecular hydration free energies by implementing the intramolecular hydrogen bond effects
Source: J Cheminform. 2015 Nov 25;7:57. doi: 10.1186/s13321-015-0106-2 (PMC4660792; doi:10.1186/s13321-015-0106-2)
Supplement: Supplementary file 1 — 10.1186/s13321-015-0106-2 Contains chemical structures, experimental and calculated solvation free energies of 763 molecules used in this study. [file 13321_2015_106_MOESM1_ESM.doc]

**SAMPL4** training set

| compound id | compound structure | $\Delta G_{sol}^{exp}$ | $\Delta G_{sol}^{calc}$ |
| --- | --- | --- | --- |
| train01 |  | 2.32 | 0.5119 |
| train02 |  | 1.23 | -0.8959 |
| train03 |  | 1.16 | 1.0752 |
| train04 |  | 1.38 | 1.0798 |
| train05 |  | 1.31 | 0.749 |
| train06 |  | 1.83 | 1.1862 |
| train07 |  | 1.31 | 1.269 |
| train08 |  | 1.01 | 0.1798 |
| train09 |  | 0.68 | 0.8392 |
| train10 |  | 0.67 | -0.2222 |
| train11 |  | -0.86 | -0.5502 |
| train12 |  | -2.4 | -1.5723 |
| train13 |  | -1.24 | -0.6465 |
| train14 |  | -0.95 | -0.3839 |
| train15 |  | -1.46 | -1.6192 |
| train16 |  | -3.35 | -2.418 |
| train17 |  | -4.57 | -5.1573 |
| train18 |  | -4.39 | -4.0098 |
| train19 |  | -4.43 | -2.0399 |
| train20 |  | -5.46 | -5.503 |
| train21 |  | -5.03 | -5.1884 |
| train22 |  | -6.92 | -6.0023 |
| train23 |  | -13.43 | -15.1752 |
| train24 |  | -6.69 | -6.6921 |
| train25 |  | -3.47 | -2.39E+00 |
| train26 |  | -6.61 | -6.3389 |
| train27 |  | -5.87 | -5.8226 |
| train28 |  | -7.66 | -7.6554 |
| train29 |  | -25.47 | -24.7518 |
| train30 |  | -4 | -3.1474 |
| train31 |  | -4.24 | -4.4007 |
| train32 |  | -4.07 | -2.7445 |
| train33 |  | -3.2 | -2.9509 |
| train34 |  | -3.22 | -3.3735 |
| train35 |  | -7.4 | -7.4285 |
| train36 |  | -7.77 | -7.6689 |
| train37 |  | -6.55 | -5.9834 |
| train38 |  | -7.17 | -5.7427 |
| train39 |  | -6.32 | -6.0238 |
| train40 |  | -5.49 | -5.4664 |
| train41 |  | -4.62 | -5.1765 |
| train42 |  | -3.45 | -4.1443 |
| train43 |  | -4.55 | -6.8864 |
| train44 |  | -7.03 | -7.3783 |
| train45 |  | -6.79 | -6.7384 |
| train46 |  | -6.06 | -5.347 |
| train47 |  | -3.18 | -2.5736 |
| train48 |  | -4.22 | -2.1781 |
| train49 |  | -3.71 | -2.9985 |
| train50 |  | -4.91 | -4.3369 |
| train51 |  | -4.02 | -4.4798 |
| train52 |  | -3.9 | -3.7182 |
| train53 |  | -5.18 | -1.0312 |
| train54 |  | -9.51 | -9.8147 |
| train55 |  | -10.47 | -10.2441 |
| train56 |  | -4.4 | -5.647 |
| train57 |  | -6.35 | -6.5757 |
| train58 |  | -8.62 | -9.0548 |
| train59 |  | -9.86 | -9.9131 |
| train60 |  | -6.27 | -6.0635 |
| train61 |  | -10.21 | -9.0873 |
| train62 |  | -10.78 | -10.6374 |
| train63 |  | -2.94 | -4.5586 |
| train64 |  | -2.36 | -2.188 |
| train65 |  | -9.63 | -9.4837 |
| train66 |  | -8.41 | -8.4698 |
| train67 |  | -5.91 | -5.0618 |
| train68 |  | -2.09 | -2.6832 |
| train69 |  | -8.18 | -8.3193 |
| train70 |  | -5.99 | -4.1893 |
| train71 |  | -6.5 | -6.4053 |
| train72 |  | -3.76 | -1.1823 |
| train73 |  | -8.9 | -13.4583 |
| train74 |  | -8.95 | -12.7414 |
| train75 |  | -3.78 | -4.8528 |
| train76 |  | -5.58 | -8.3142 |
| train77 |  | -7.8 | -8.4537 |

**SAMPL4 test** set

| compound id | compound structure | $\Delta G_{sol}^{exp}$ | $\Delta G_{sol}^{calc}$ |
| --- | --- | --- | --- |
| SAMPL4_001 |  | -23.62 | -28.0144 |
| SAMPL4_002 |  | -2.49 | -1.3438 |
| SAMPL4_003 |  | -4.78 | -3.9572 |
| SAMPL4_004 |  | -4.45 | -3.9476 |
| SAMPL4_005 |  | -5.33 | -4.0576 |
| SAMPL4_006 |  | -5.26 | -7.5645 |
| SAMPL4_009 |  | -8.24 | -13.5367 |
| SAMPL4_010 |  | -6.24 | -6.9314 |
| SAMPL4_011 |  | -7.78 | -12.6857 |
| SAMPL4_012 |  | -3.75 | -2.6681 |
| SAMPL4_013 |  | -4.44 | -3.6921 |
| SAMPL4_014 |  | -4.09 | -2.3601 |
| SAMPL4_015 |  | -4.51 | -0.9817 |
| SAMPL4_016 |  | -3.2 | -3.1912 |
| SAMPL4_017 |  | -2.53 | -2.2069 |
| SAMPL4_019 |  | -3.78 | -2.4537 |
| SAMPL4_020 |  | -2.78 | -1.4905 |
| SAMPL4_021 |  | -7.63 | -9.1157 |
| SAMPL4_022 |  | -6.78 | -11.2788 |
| SAMPL4_023 |  | -9.34 | -5.8035 |
| SAMPL4_024 |  | -7.43 | -5.4342 |
| SAMPL4_025 |  | -5.73 | -5.73E+00 |
| SAMPL4_026 |  | -5.31 | -4.5446 |
| SAMPL4_027 |  | -4.8 | -6.0553 |
| SAMPL4_028 |  | -4.29 | -5.2005 |
| SAMPL4_029 |  | -1.66 | -3.0335 |
| SAMPL4_030 |  | -2.29 | -3.2338 |
| SAMPL4_032 |  | -7.29 | -7.5327 |
| SAMPL4_033 |  | -6.96 | -9.0346 |
| SAMPL4_034 |  | -5.8 | -7.542 |
| SAMPL4_035 |  | -4.68 | -9.4128 |
| SAMPL4_036 |  | -5.66 | -5.8408 |
| SAMPL4_037 |  | -5.94 | -8.0751 |
| SAMPL4_038 |  | -3.93 | -4.2901 |
| SAMPL4_039 |  | -0.85 | -0.3613 |
| SAMPL4_041 |  | -5.05 | -4.2537 |
| SAMPL4_042 |  | -3.13 | -2.4482 |
| SAMPL4_043 |  | -0.14 | -0.6982 |
| SAMPL4_044 |  | -5.08 | -4.0379 |
| SAMPL4_045 |  | -11.53 | -13.2238 |
| SAMPL4_046 |  | -9.44 | -13.3352 |
| SAMPL4_047 |  | -14.21 | -17.6057 |
| SAMPL4_048 |  | -11.85 | -17.1259 |
| SAMPL4_049 |  | -3.16 | -5.6722 |
| SAMPL4_050 |  | -4.14 | -2.416 |
| SAMPL4_051 |  | -9.53 | -17.0332 |
| SAMPL4_052 |  | -2.87 | -3.4018 |

**FSD training set**

| compound id | compound structure | $\Delta G_{sol}^{exp}$ | $\Delta G_{sol}^{calc}$ |
| --- | --- | --- | --- |
| mobley_1017962 |  | -2.49 | -2.4547 |
| mobley_1034539 |  | -3.04 | -3.0447 |
| mobley_1075836 |  | -2.93 | -3.4636 |
| mobley_1079207 |  | -0.98 | -2.102 |
| mobley_1107178 |  | -0.74 | -0.7703 |
| mobley_1178614 |  | -4.55 | -6.8047 |
| mobley_1199854 |  | -0.53 | -0.3362 |
| mobley_1231151 |  | -0.25 | 0.309 |
| mobley_1261349 |  | 2.51 | 1.2076 |
| mobley_129464 |  | -0.83 | 0.0251 |
| mobley_1328465 |  | -1.99 | -0.7517 |
| mobley_1396156 |  | -5.22 | -3.4909 |
| mobley_1417007 |  | -9.01 | -9.2879 |
| mobley_1449384 |  | -0.68 | -0.2868 |
| mobley_1502181 |  | -2.49 | -2.7434 |
| mobley_1520842 |  | -4.93 | -3.8255 |
| mobley_1571523 |  | -6.16 | -5.4118 |
| mobley_1592519 |  | -2.23 | -2.2657 |
| mobley_1615431 |  | -0.53 | 0.3269 |
| mobley_1636752 |  | -5.1 | -5.5971 |
| mobley_1708457 |  | -10.08 | -4.5231 |
| mobley_1722522 |  | -2.49 | -2.5804 |
| mobley_1728386 |  | -11 | -1.07E+01 |
| mobley_1733799 |  | -5.85 | -7.1085 |
| mobley_1743409 |  | -5.71 | -5.5628 |
| mobley_1755375 |  | -5.26 | -5.3908 |
| mobley_1760914 |  | -1.14 | -1.5379 |
| mobley_1781152 |  | -2.4 | -2.1007 |
| mobley_1792062 |  | -2.33 | -1.4266 |
| mobley_1821184 |  | -5.88 | -3.1142 |
| mobley_1827204 |  | -2.81 | -2.2396 |
| mobley_1838110 |  | 1.77 | 0.4357 |
| mobley_1857976 |  | -1.79 | -0.6151 |
| mobley_186894 |  | -1.1 | -0.6355 |
| mobley_1873346 |  | -0.9 | -1.4499 |
| mobley_1875719 |  | -3.41 | -3.2988 |
| mobley_1881249 |  | -3.52 | -3.3472 |
| mobley_1896013 |  | -20.52 | -19.7696 |
| mobley_1899443 |  | 1.47 | 1.1699 |
| mobley_1903702 |  | -4.62 | -4.1561 |
| mobley_1922649 |  | -7.19 | -6.9623 |
| mobley_1923244 |  | 2.1 | 0.6115 |
| mobley_194273 |  | -9.73 | -9.624 |
| mobley_1944394 |  | -9.34 | -7.4134 |
| mobley_1952272 |  | -4.02 | -2.0222 |
| mobley_1963873 |  | -10 | -7.9636 |
| mobley_1976156 |  | -2.07 | -1.8332 |
| mobley_2008055 |  | 1.83 | 0.365 |
| mobley_2043882 |  | -3.9 | -2.7874 |
| mobley_20524 |  | -6.6 | -6.9513 |
| mobley_2068538 |  | 2 | 0.1966 |
| mobley_2099370 |  | -10.78 | -11.2313 |
| mobley_2123854 |  | -10.17 | -10.1308 |
| mobley_2126135 |  | -5.66 | -5.944 |
| mobley_2143011 |  | 1.83 | 1.1018 |
| mobley_2178600 |  | -0.1 | 0.2599 |
| mobley_2183616 |  | -0.45 | -0.7689 |
| mobley_2197088 |  | 3.16 | 1.4 |
| mobley_2198613 |  | -0.63 | -0.1402 |
| mobley_2245668 |  | -4.5 | -4.4945 |
| mobley_2261979 |  | -3.15 | -1.6173 |
| mobley_2269032 |  | -10.21 | -7.6473 |
| mobley_2295058 |  | -9.51 | -9.4648 |
| mobley_2310185 |  | -5 | -5.0566 |
| mobley_2316618 |  | -14.21 | -14.9962 |
| mobley_2328633 |  | 2.55 | 1.542 |
| mobley_2341732 |  | -2.56 | -2.5999 |
| mobley_2364370 |  | -2.64 | -2.7565 |
| mobley_2371092 |  | -5.22 | -3.1214 |
| mobley_2390199 |  | -2.92 | -2.8885 |
| mobley_2402487 |  | -6 | -6.0423 |
| mobley_2410897 |  | -4.5 | -4.4326 |
| mobley_2422586 |  | -1.66 | -0.5571 |
| mobley_2481002 |  | -1.45 | -2.7556 |
| mobley_2484519 |  | -1.93 | -1.7042 |
| mobley_2487143 |  | -3.28 | -3.1734 |
| mobley_2492140 |  | -1.23 | -1.2053 |
| mobley_2523689 |  | -16.43 | -15.6722 |
| mobley_252413 |  | 2.88 | 1.4727 |
| mobley_2577969 |  | -4.52 | -2.9793 |
| mobley_2613240 |  | -5.33 | -3.0704 |
| mobley_2636578 |  | -4.8 | -6.1726 |
| mobley_2681549 |  | -0.78 | -2.0385 |
| mobley_2689721 |  | 1.23 | 0.6908 |
| mobley_2725802 |  | -9.8 | -5.6276 |
| mobley_2751110 |  | -10.64 | -8.433 |
| mobley_2771569 |  | -7.77 | -7.6649 |
| mobley_2782339 |  | -9.61 | -9.93E+00 |
| mobley_2784376 |  | 0.75 | 0.3102 |
| mobley_2789243 |  | -4.01 | -4.3973 |
| mobley_2792521 |  | -3.68 | -3.1946 |
| mobley_2802855 |  | -0.4 | -0.5411 |
| mobley_282648 |  | -2.4 | -2.7343 |
| mobley_2837389 |  | -4.78 | -3.4573 |
| mobley_2844990 |  | -3.84 | -4.3532 |
| mobley_2845466 |  | -3.37 | -4.3544 |
| mobley_2864987 |  | -4.39 | -4.1009 |
| mobley_2913224 |  | -9.94 | -10.4588 |
| mobley_2923700 |  | -6.13 | -5.9733 |
| mobley_2925352 |  | -6.13 | -6.1827 |
| mobley_2929847 |  | -6.09 | -5.7016 |
| mobley_2960202 |  | -3.71 | -4.2693 |
| mobley_296847 |  | -4.69 | -4.5596 |
| mobley_2972345 |  | -3.78 | -2.2399 |
| mobley_2972906 |  | -1.4 | -1.5055 |
| mobley_299266 |  | 1.16 | 0.8959 |
| mobley_2996632 |  | -1.08 | -0.8873 |
| mobley_303222 |  | 1.32 | 0.5823 |
| mobley_3034976 |  | -6.69 | -7.076 |
| mobley_3105103 |  | -11.14 | -9.3809 |
| mobley_313406 |  | -4.85 | -5.1089 |
| mobley_3151666 |  | -3.64 | -4.1074 |
| mobley_3167746 |  | 2.88 | 1.1353 |
| mobley_3201701 |  | -9.41 | -11.008 |
| mobley_3234716 |  | -0.8 | -0.9024 |
| mobley_3264884 |  | -7.47 | -7.2505 |
| mobley_3265457 |  | -7.78 | -9.5707 |
| mobley_3266352 |  | -1.92 | -2.5815 |
| mobley_3269565 |  | -2.01 | -0.2542 |
| mobley_3274817 |  | -6.23 | -7.7128 |
| mobley_3318135 |  | -2.64 | -2.5427 |
| mobley_3359593 |  | -5.26 | -6.0006 |
| mobley_3370989 |  | -0.11 | 1.13 |
| mobley_337666 |  | -16.92 | -16.702 |
| mobley_3378420 |  | -3.88 | -4.2093 |
| mobley_3398536 |  | -1.74 | -2.7962 |
| mobley_3414356 |  | -3.92 | -3.6896 |
| mobley_3452749 |  | -1.21 | -0.3099 |
| mobley_3515580 |  | -5.94 | -7.2493 |
| mobley_352111 |  | -6.34 | -6.2909 |
| mobley_3525176 |  | -0.23 | -0.926 |
| mobley_3546460 |  | -4.4 | -4.5925 |
| mobley_3572203 |  | -2.11 | -2.33 |
| mobley_3573480 |  | -3.04 | -3.0223 |
| mobley_3589456 |  | -4.59 | -1.8942 |
| mobley_36119 |  | -4.09 | -4.3535 |
| mobley_3639400 |  | -0.16 | 0.1277 |
| mobley_3663158 |  | -8.11 | -7.2356 |
| mobley_3686115 |  | -3.28 | -0.8862 |
| mobley_3690931 |  | -2.28 | -2.9956 |
| mobley_3709920 |  | 2.06 | 1.3926 |
| mobley_3715043 |  | -2.82 | -1.3387 |
| mobley_3727287 |  | -6.5 | -9.2109 |
| mobley_3738859 |  | -0.1 | -0.1445 |
| mobley_3746675 |  | -1.24 | -0.9615 |
| mobley_3761215 |  | -0.19 | -0.5785 |
| mobley_3762186 |  | -1.31 | -0.7673 |
| mobley_3775790 |  | -2.89 | -4.1004 |
| mobley_3802803 |  | -1.66 | -2.4469 |
| mobley_3843583 |  | -7.28 | -6.9323 |
| mobley_3867265 |  | -3.8 | -3.866 |
| mobley_3968043 |  | -4.7 | -3.5516 |
| mobley_3968739 |  | -3.92 | -4.9034 |
| mobley_3969312 |  | -4.02 | -4.6801 |
| mobley_3980099 |  | -1.24 | -2.1087 |
| mobley_3982371 |  | -3.13 | -3.2442 |
| mobley_4013838 |  | -2.74 | -2.2088 |
| mobley_4035953 |  | -5.9 | -6.1438 |
| mobley_4039055 |  | -18.17 | -16.8573 |
| mobley_4043951 |  | 1.68 | 0.8127 |
| mobley_4043987 |  | 2.93 | 1.3078 |
| mobley_4149784 |  | -3.67 | -4.4557 |
| mobley_4177472 |  | 2.56 | 1.9692 |
| mobley_4188615 |  | -9.29 | -7.3205 |
| mobley_4219614 |  | -1.34 | -2.0851 |
| mobley_4252724 |  | 0.16 | -0.6829 |
| mobley_4287564 |  | -4.63 | -3.8809 |
| mobley_4291494 |  | -4.39 | -4.4076 |
| mobley_430089 |  | 0.01 | -0.1431 |
| mobley_4434915 |  | -0.55 | -0.21 |
| mobley_4463913 |  | -4.97 | -5.9434 |
| mobley_4465023 |  | -1.17 | -1.4295 |
| mobley_4479135 |  | -2.78 | -2.7736 |
| mobley_4483973 |  | -0.8 | -1.5154 |
| mobley_4494568 |  | -1.39 | -1.8873 |
| mobley_4506634 |  | -0.11 | 0.3337 |
| mobley_4561957 |  | 2.34 | 1.4405 |
| mobley_4587267 |  | -23.62 | -28.4998 |
| mobley_4603202 |  | -4.23 | -1.4805 |
| mobley_4609460 |  | -9.2 | -7.9977 |
| mobley_4620651 |  | -4.1 | -2.7266 |
| mobley_4630641 |  | -4.84 | -1.6767 |
| mobley_4678740 |  | 1.07 | 1.105 |
| mobley_4687447 |  | -6.4 | -5.8338 |
| mobley_468867 |  | -2.55 | -1.3255 |
| mobley_4690963 |  | -3.54 | -1.3485 |
| mobley_4694328 |  | -2.29 | -1.9647 |
| mobley_4699732 |  | -1.27 | -0.1061 |
| mobley_4715906 |  | 1.58 | 1.4947 |
| mobley_4762983 |  | 1.66 | 1.1298 |
| mobley_4780078 |  | -6.01 | -5.3909 |
| mobley_4845722 |  | -2.33 | -2.07E+00 |
| mobley_4850657 |  | -4.87 | -4.875 |
| mobley_486214 |  | -6.96 | -7.5214 |
| mobley_4883284 |  | -5.49 | -6.9267 |
| mobley_4884177 |  | -4.59 | -3.5795 |
| mobley_4893032 |  | -3.52 | -3.1447 |
| mobley_4924862 |  | -2.13 | -3.0161 |
| mobley_49274 |  | 0.6 | 0.1229 |
| mobley_4934872 |  | -5.23 | -3.47 |
| mobley_4936555 |  | -6.78 | -7.8112 |
| mobley_4964807 |  | -5.99 | -5.9942 |
| mobley_4983965 |  | -1.46 | -0.6334 |
| mobley_5003962 |  | -11.53 | -14.3046 |
| mobley_5026370 |  | -6.02 | -7.7632 |
| mobley_5052949 |  | -1.28 | -0.3887 |
| mobley_5056289 |  | -3.3 | -0.2779 |
| mobley_5063386 |  | -4.58 | -8.4279 |
| mobley_5072416 |  | -6.12 | -7.0448 |
| mobley_5094777 |  | -1.46 | -0.9744 |
| mobley_5110043 |  | -2.63 | -1.3938 |
| mobley_5123639 |  | -3.73 | -3.4712 |
| mobley_5157661 |  | 2.3 | 0.9057 |
| mobley_5200358 |  | -9.84 | -10.9581 |
| mobley_5220185 |  | -5.45 | -5.894 |
| mobley_525934 |  | -1.2 | -0.8493 |
| mobley_5263791 |  | -2.4 | -1.8658 |
| mobley_52782 |  | -5.73 | -6.2751 |
| mobley_5282042 |  | -7.43 | -6.0323 |
| mobley_5286200 |  | -2.32 | -0.178 |
| mobley_5310099 |  | 1.58 | 0.9943 |
| mobley_5311804 |  | -6.62 | -6.6291 |
| mobley_5326154 |  | -3.45 | -3.9319 |
| mobley_5346580 |  | -3.11 | -2.6275 |
| mobley_5371840 |  | -3.1 | -4.5625 |
| mobley_5393242 |  | -6.48 | -6.8245 |
| mobley_5456566 |  | -2.98 | -3.0233 |
| mobley_5467162 |  | -5.91 | -5.1756 |
| mobley_5471704 |  | -2.21 | -2.2494 |
| mobley_547634 |  | -2.78 | -1.4401 |
| mobley_5494918 |  | 2.93 | 1.8798 |
| mobley_5499659 |  | -8.41 | -6.3089 |
| mobley_550662 |  | -4.42 | -1.7973 |
| mobley_5510474 |  | -3.81 | -4.4105 |
| mobley_5518547 |  | -5.57 | -6.1789 |
| mobley_5520946 |  | -2.55 | -2.6484 |
| mobley_5561855 |  | -1.96 | -2.9982 |
| mobley_5571660 |  | -5.21 | -5.0438 |
| mobley_5627459 |  | -2.86 | -2.2598 |
| mobley_5665561 |  | -2.47 | -1.3638 |
| mobley_5690766 |  | 1.01 | 1.1 |
| mobley_5708811 |  | -9.86 | -7.7821 |
| mobley_5747188 |  | -3.58 | -3.1691 |
| mobley_5759258 |  | -4.57 | -4.7431 |
| mobley_5760563 |  | -5.1 | -6.6089 |
| mobley_5816127 |  | -6.62 | -6.1964 |
| mobley_5852491 |  | 1.59 | 0.9754 |
| mobley_588781 |  | -0.04 | -0.7878 |
| mobley_5890803 |  | -2.44 | -2.612 |
| mobley_5917842 |  | -5.8 | -6.4373 |
| mobley_5935995 |  | 2.51 | 1.3344 |
| mobley_5948990 |  | -7.37 | -8.2503 |
| mobley_5977084 |  | -4.77 | -3.7825 |
| mobley_6006813 |  | -3.09 | -1.7892 |
| mobley_6060301 |  | -3.95 | -3.8251 |
| mobley_6081058 |  | -3.43 | -2.7809 |
| mobley_6082662 |  | -9.82 | -8.4331 |
| mobley_6091882 |  | 1.28 | 1.4562 |
| mobley_6102880 |  | -2.87 | -4.1456 |
| mobley_6115639 |  | -8.7 | -6.039 |
| mobley_6190089 |  | -2.37 | -0.9676 |
| mobley_6195751 |  | -8.68 | -9.313 |
| mobley_6198745 |  | -8.84 | -8.2832 |
| mobley_6201330 |  | -9.37 | -9.084 |
| mobley_6232400 |  | -7.1 | -6.8638 |
| mobley_6235784 |  | 0 | 0.4969 |
| mobley_6239320 |  | -18.06 | -26.1186 |
| mobley_6248915 |  | -3.65 | -3.5629 |
| mobley_6250025 |  | 0.08 | -0.2172 |
| mobley_6266306 |  | -5.56 | -4.3462 |
| mobley_627267 |  | -0.03 | 5.29E-03 |
| mobley_628086 |  | -4.16 | -3.4117 |
| mobley_628951 |  | -2.28 | -3.0501 |
| mobley_6303022 |  | -4.73 | -3.6286 |
| mobley_6334915 |  | -12.74 | -9.2181 |
| mobley_6338073 |  | -6.68 | -9.4353 |
| mobley_6353617 |  | -8.26 | -7.5963 |
| mobley_6358463 |  | 1.07 | 1.0207 |
| mobley_6359135 |  | 0.08 | -1.195 |
| mobley_6359156 |  | -2.46 | -3.1478 |
| mobley_63712 |  | -3.88 | -4.3707 |
| mobley_637522 |  | -3.95 | -3.1994 |
| mobley_6430250 |  | -0.53 | -1.208 |
| mobley_6456034 |  | -1.69 | -1.609 |
| mobley_646007 |  | -5.48 | -4.0919 |
| mobley_6497672 |  | -4.22 | -3.4621 |
| mobley_6522117 |  | -7.07 | -7.0457 |
| mobley_6571751 |  | 0.67 | 0.2669 |
| mobley_6619554 |  | -2.49 | -2.411 |
| mobley_664966 |  | -2.49 | -2.0393 |
| mobley_6714389 |  | -4.55 | -4.8063 |
| mobley_6727159 |  | -8.18 | -8.5255 |
| mobley_6739648 |  | -5.06 | -1.9215 |
| mobley_6743808 |  | -6.5 | -5.4421 |
| mobley_676247 |  | 3.12 | 1.6141 |
| mobley_6794076 |  | -7.62 | -7.6761 |
| mobley_6804509 |  | -1.34 | -2.0726 |
| mobley_6854178 |  | -2.02 | -3.3716 |
| mobley_6896128 |  | 2.97 | 1.4512 |
| mobley_6911232 |  | -2.1 | -0.6977 |
| mobley_6917738 |  | -6.55 | -5.4333 |
| mobley_6929123 |  | -4 | -3.3827 |
| mobley_6973347 |  | -2.94 | -3.0598 |
| mobley_6981465 |  | -2.64 | -2.5321 |
| mobley_6988468 |  | -4.33 | -3.6919 |
| mobley_7009711 |  | -6.27 | -5.2684 |
| mobley_7010316 |  | -5.46 | -4.2681 |
| mobley_7015518 |  | -1.91 | -0.8622 |
| mobley_7017274 |  | -3.88 | -3.0782 |
| mobley_7039935 |  | -7.67 | -7.0337 |
| mobley_7047032 |  | 1.09 | 0.594 |
| mobley_7066554 |  | -7.03 | -6.7847 |
| mobley_7099614 |  | -0.57 | 0.0738 |
| mobley_7106722 |  | 2.51 | 1.1717 |
| mobley_7142697 |  | -4.09 | -3.9746 |
| mobley_7150646 |  | -2.3 | -2.865 |
| mobley_7157427 |  | 0.29 | 0.5242 |
| mobley_7176248 |  | -3.25 | -3.4127 |
| mobley_718988 |  | -3.79 | -3.6881 |
| mobley_7200804 |  | -5.29 | -6.0852 |
| mobley_7239499 |  | -3.52 | -4.591 |
| mobley_7295828 |  | -2.45 | -2.5992 |
| mobley_7298388 |  | -3.45 | -3.1284 |
| mobley_7326706 |  | -5.04 | -5.0345 |
| mobley_7326982 |  | -4.74 | -4.5908 |
| mobley_7360181 |  | -0.8 | -0.8322 |
| mobley_7364468 |  | -5.18 | -2.7446 |
| mobley_7378987 |  | -12.64 | -15.9679 |
| mobley_7393673 |  | -5.33 | -5.1926 |
| mobley_7417968 |  | -5.31 | -3.8285 |
| mobley_7455579 |  | -2.22 | -3.1045 |
| mobley_7463408 |  | -7.78 | -8.5343 |
| mobley_7463799 |  | -6.92 | -6.5005 |
| mobley_7532833 |  | -3.88 | -3.6662 |
| mobley_755351 |  | -7.29 | -7.088 |
| mobley_7578802 |  | -2.13 | -2.1328 |
| mobley_7608462 |  | -1.12 | -2.1693 |
| mobley_7610437 |  | -3.88 | -3.3635 |
| mobley_7688753 |  | -9.52 | -9.1621 |
| mobley_7690440 |  | -1.83 | -2.4 |
| mobley_7732703 |  | 1.79 | 0.4981 |
| mobley_7758918 |  | -6.46 | -6.6116 |
| mobley_7768165 |  | -1.95 | -1.0247 |
| mobley_7769613 |  | 0.84 | 0.1813 |
| mobley_7774695 |  | -4.69 | -3.3506 |
| mobley_778352 |  | 1.31 | 0.105 |
| mobley_7794077 |  | -15.46 | -15.3437 |
| mobley_7814642 |  | -1.12 | -2.0878 |
| mobley_7859387 |  | -2.67 | -2.0938 |
| mobley_7869158 |  | -1.82 | -2.3027 |
| mobley_7912193 |  | -8.72 | -8.9363 |
| mobley_7977115 |  | -4.06 | -3.8247 |
| mobley_7983227 |  | -4.42 | -4.1548 |
| mobley_7988076 |  | -8.83 | -9.2472 |
| mobley_8006582 |  | 1.2 | 0.5579 |
| mobley_8011706 |  | -7.81 | -5.3238 |
| mobley_8048190 |  | -9.71 | -9.6161 |
| mobley_8052240 |  | -9.44 | -13.4969 |
| mobley_8117218 |  | -4.82 | -0.7493 |
| mobley_8118832 |  | -5.03 | -4.7535 |
| mobley_8124669 |  | -8.21 | -5.5778 |
| mobley_8191186 |  | 0.06 | 0.9851 |
| mobley_8207196 |  | -10.22 | -10.2147 |
| mobley_820789 |  | -6.35 | -6.373 |
| mobley_8208692 |  | -4.61 | -2.9795 |
| mobley_8221999 |  | -3.64 | -3.2945 |
| mobley_8260524 |  | -0.48 | -0.4219 |
| mobley_8311321 |  | -0.5 | 0.5339 |
| mobley_8337722 |  | -3.2 | -2.3009 |
| mobley_8337977 |  | -3.05 | -2.8083 |
| mobley_8427539 |  | -9.4 | -8.8778 |
| mobley_8449031 |  | -3.22 | -3.99 |
| mobley_8492526 |  | -3.03 | -2.3804 |
| mobley_8514745 |  | -2.51 | -2.6174 |
| mobley_8525830 |  | -0.14 | -0.3517 |
| mobley_852937 |  | -6.19 | -4.9645 |
| mobley_8558116 |  | -6.32 | -5.6856 |
| mobley_8573194 |  | -3.18 | -2.54 |
| mobley_859464 |  | -2.36 | -2.4143 |
| mobley_8614858 |  | 2.67 | 1.0028 |
| mobley_8668219 |  | 2.52 | 1.5837 |
| mobley_8685905 |  | 0.5 | 1.0285 |
| mobley_8705848 |  | -3.22 | -2.5308 |
| mobley_8713762 |  | -4.13 | -5.4438 |
| mobley_8723116 |  | -5.9 | -5.3908 |
| mobley_8739734 |  | -3.24 | -3.06 |
| mobley_8746821 |  | -4.82 | -3.2046 |
| mobley_8754702 |  | -2.48 | -3.6711 |
| mobley_8764620 |  | -6.75 | -7.7029 |
| mobley_8765203 |  | 0.68 | 1.1778 |
| mobley_8772587 |  | -0.25 | -0.4866 |
| mobley_8785107 |  | -2.92 | 0.1018 |
| mobley_8789465 |  | -6.2 | -6.9382 |
| mobley_8798016 |  | -7 | -6.9279 |
| mobley_8809190 |  | -0.4 | -1.0619 |
| mobley_8809274 |  | -0.25 | -0.4837 |
| mobley_8823527 |  | -3.61 | -3.0333 |
| mobley_8861672 |  | -4.86 | -3.145 |
| mobley_8883511 |  | -3.93 | -4.0661 |
| mobley_8885088 |  | 0.56 | -0.1701 |
| mobley_8899867 |  | -5.49 | -4.4677 |
| mobley_8966374 |  | -5.51 | -6.1053 |
| mobley_8983100 |  | -0.82 | -0.6299 |
| mobley_9015240 |  | 0.52 | 0.2543 |
| mobley_9028462 |  | -1.1 | -1.9928 |
| mobley_902954 |  | -2.09 | -2.7217 |
| mobley_9029594 |  | -0.22 | 0.5169 |
| mobley_9073553 |  | -1.61 | -1.1078 |
| mobley_9112978 |  | 1.31 | -0.1086 |
| mobley_9114381 |  | -6.72 | -6.646 |
| mobley_9139060 |  | 2.11 | 1.5006 |
| mobley_9185328 |  | -4.39 | -4.3115 |
| mobley_9201263 |  | -4.87 | -5.5459 |
| mobley_9246215 |  | 0.93 | 0.9022 |
| mobley_9257453 |  | -7.29 | -6.6335 |
| mobley_9407874 |  | -2.88 | -2.8793 |
| mobley_9414831 |  | -2.44 | -2.0215 |
| mobley_9434451 |  | -0.48 | -0.114 |
| mobley_9460824 |  | -4.37 | -4.4834 |
| mobley_9478823 |  | -0.9 | -1.0261 |
| mobley_9510785 |  | -3.48 | -3.1132 |
| mobley_951560 |  | -7.4 | -7.3779 |
| mobley_9557440 |  | -17.74 | -16.9712 |
| mobley_9624458 |  | -1.89 | -0.4762 |
| mobley_9626434 |  | -6.69 | -5.9882 |
| mobley_967099 |  | -3.13 | -1.3453 |
| mobley_9671033 |  | -3.34 | -1.6511 |
| mobley_9717937 |  | -2.69 | -3.2178 |
| mobley_9729792 |  | -0.99 | -1.606 |
| mobley_9733743 |  | -4.24 | -4.1322 |
| mobley_9740891 |  | -4.71 | -5.0047 |
| mobley_9794857 |  | -2.21 | 0.145 |
| mobley_9821936 |  | -4.53 | -4.2009 |
| mobley_9838013 |  | -4.43 | -3.9459 |
| mobley_9897248 |  | -4.78 | -4.5829 |
| mobley_9913368 |  | -0.78 | -1.4746 |
| mobley_9942801 |  | -0.95 | -0.7331 |
| mobley_994483 |  | -0.74 | -0.5643 |
| mobley_9974966 |  | -1.21 | 0.1002 |

**FSD test set**

| compound id | compound structure | $\Delta G_{sol}^{exp}$ | $\Delta G_{sol}^{calc}$ |
| --- | --- | --- | --- |
| mobley_1019269 |  | -4.72 | -4.7099 |
| mobley_1036761 |  | -4.59 | -3.3007 |
| mobley_1046331 |  | -3.82 | -4.0331 |
| mobley_1139153 |  | 2.89 | 1.947 |
| mobley_1144156 |  | -1.59 | -0.5765 |
| mobley_1160109 |  | -3.75 | -1.7791 |
| mobley_1189457 |  | -2.73 | -2.8873 |
| mobley_1235151 |  | -2.82 | -1.2247 |
| mobley_1244778 |  | -5.48 | -3.7676 |
| mobley_1278715 |  | -0.64 | -1.405 |
| mobley_1323538 |  | -7.5 | -4.9935 |
| mobley_1328936 |  | -6.88 | -6.4538 |
| mobley_1352110 |  | -3.64 | -5.3981 |
| mobley_1363784 |  | -4.84 | -3.1465 |
| mobley_1424265 |  | -0.83 | -0.8827 |
| mobley_1469079 |  | -3.56 | -4.5534 |
| mobley_1527293 |  | -8.42 | -8.2904 |
| mobley_1563176 |  | -0.99 | -0.3748 |
| mobley_1650157 |  | -4.47 | -4.1049 |
| mobley_1659169 |  | -7.17 | -4.0977 |
| mobley_1662128 |  | 1.38 | 0.742 |
| mobley_1674094 |  | 0.29 | -1.11E-03 |
| mobley_1717215 |  | -2.78 | -2.3115 |
| mobley_1723043 |  | 3.43 | 4.0416 |
| mobley_172879 |  | -3.15 | -4.7348 |
| mobley_1735893 |  | -6.21 | -5.9756 |
| mobley_1770205 |  | -10.03 | -17.391 |
| mobley_1800170 |  | -1.14 | -0.6163 |
| mobley_1803862 |  | 2.38 | 1.0522 |
| mobley_1849020 |  | -6.5 | -6.2017 |
| mobley_1855337 |  | -1.62 | -2.0426 |
| mobley_1858644 |  | -6.79 | -6.4625 |
| mobley_1893815 |  | -1.43 | -1.0039 |
| mobley_1893937 |  | 0 | 0.4895 |
| mobley_1905088 |  | -2.38 | -2.4283 |
| mobley_1967551 |  | -3.5 | -3.0491 |
| mobley_197466 |  | -4.61 | -3.2546 |
| mobley_1977493 |  | -0.33 | 0.0817 |
| mobley_1987439 |  | -0.9 | -0.218 |
| mobley_2005792 |  | -2.7 | -3.4386 |
| mobley_2023925 |  | 0.1 | 0.9529 |
| mobley_2049967 |  | -1.5 | -0.8706 |
| mobley_2078467 |  | -7 | -5.9376 |
| mobley_210639 |  | -0.46 | -0.3305 |
| mobley_2146331 |  | -2.75 | -3.4384 |
| mobley_2213823 |  | 2.51 | 1.1968 |
| mobley_2279874 |  | -4.42 | -2.4838 |
| mobley_2294995 |  | -2.56 | -2.1645 |
| mobley_2354112 |  | -6.25 | -6.0119 |
| mobley_242480 |  | -11.85 | -18.5036 |
| mobley_2451097 |  | -4.1 | -5.3611 |
| mobley_2457863 |  | -7.66 | -7.3645 |
| mobley_2489709 |  | -1.36 | -2.1461 |
| mobley_2493732 |  | 0.25 | -0.2917 |
| mobley_2501588 |  | -2.45 | -3.5991 |
| mobley_2517158 |  | 0.34 | 0.1408 |
| mobley_2518989 |  | -5.74 | -11.9091 |
| mobley_2607611 |  | -2.26 | -2.4468 |
| mobley_2609604 |  | 2.56 | 1.5397 |
| mobley_2659552 |  | -9.76 | -6.665 |
| mobley_2661134 |  | -9.65 | -9.9636 |
| mobley_2693089 |  | -5.04 | -5.0161 |
| mobley_2725215 |  | -7.98 | -8.9691 |
| mobley_2727678 |  | -18.72 | -14.1986 |
| mobley_2763835 |  | -2.22 | -2.4342 |
| mobley_2850833 |  | -4.68 | -9.1823 |
| mobley_2859600 |  | -1.24 | -1.2197 |
| mobley_2881590 |  | -1.88 | -2.1842 |
| mobley_2958326 |  | -3.65 | -3.3182 |
| mobley_3006808 |  | -4.31 | -3.9462 |
| mobley_3040612 |  | -0.85 | -0.6645 |
| mobley_3047364 |  | -7.65 | -10.1428 |
| mobley_3053621 |  | -0.9 | -2.3042 |
| mobley_3060426 |  | 2.93 | 2.1093 |
| mobley_3083321 |  | -2.93 | -1.7113 |
| mobley_3144334 |  | -5.91 | -5.6179 |
| mobley_3169935 |  | -4.91 | -6.7087 |
| mobley_3183805 |  | -0.86 | -0.2414 |
| mobley_3187514 |  | -5.53 | -6.0871 |
| mobley_3210206 |  | -4.95 | -5.6403 |
| mobley_3211679 |  | 0.14 | -0.0791 |
| mobley_3259411 |  | -13.43 | -15.0439 |
| mobley_3269819 |  | -3.17 | -3.1107 |
| mobley_3323117 |  | -8.61 | -7.3131 |
| mobley_3325209 |  | -4.04 | -3.2747 |
| mobley_3395921 |  | -3.12 | -0.5422 |
| mobley_3425174 |  | -0.77 | -5.94E-03 |
| mobley_349850 |  | -4.7 | -4.5651 |
| mobley_3682850 |  | -4.91 | -3.3968 |
| mobley_3777264 |  | -8.84 | -8.3924 |
| mobley_397645 |  | -3.64 | -4.6263 |
| mobley_3976574 |  | -4.15 | -4.3145 |
| mobley_3999471 |  | -0.84 | -0.2411 |
| mobley_4059279 |  | -7.58 | -8.9208 |
| mobley_4193752 |  | -4.12 | -3.9165 |
| mobley_4218209 |  | -7.48 | -7.2108 |
| mobley_4305650 |  | -3.84 | -3.2829 |
| mobley_4338603 |  | -15.83 | -16.3105 |
| mobley_4364398 |  | -0.89 | -0.8873 |
| mobley_4371692 |  | -9.53 | -18.6389 |
| mobley_4375719 |  | 3.13 | 1.3025 |
| mobley_4395315 |  | -6.79 | -4.9104 |
| mobley_4553008 |  | -1.01 | -2.0485 |
| mobley_4584540 |  | -4.59 | -3.0879 |
| mobley_4613090 |  | -7.63 | -7.4222 |
| mobley_4639255 |  | -9.3 | -10.4899 |
| mobley_4683624 |  | -5.21 | -5.8403 |
| mobley_4689084 |  | -6.34 | -5.9208 |
| mobley_4759887 |  | 0.27 | -0.0517 |
| mobley_4792268 |  | -6.16 | -6.1155 |
| mobley_5006685 |  | -4.09 | -1.8291 |
| mobley_5076071 |  | -5.66 | -8.3573 |
| mobley_5079234 |  | -4.21 | -4.2857 |
| mobley_511661 |  | 0.56 | 0.9018 |
| mobley_5347550 |  | -1.12 | -0.461 |
| mobley_5390332 |  | -2.83 | -2.8552 |
| mobley_5445548 |  | -0.3 | -0.88 |
| mobley_5449201 |  | 2.3 | 0.7709 |
| mobley_5538249 |  | -2.16 | -3.0909 |
| mobley_5600967 |  | -11.01 | -7.7187 |
| mobley_5616693 |  | -2.53 | -1.6323 |
| mobley_5692472 |  | -4.29 | -3.5482 |
| mobley_5732611 |  | -10.27 | -4.9497 |
| mobley_5747981 |  | -5.73 | -2.7457 |
| mobley_5857 |  | -5.72 | -4.8063 |
| mobley_5880265 |  | -6.25 | -5.2331 |
| mobley_590519 |  | -1.64 | -1.8453 |
| mobley_5952846 |  | 0.1 | -1.8442 |
| mobley_5973402 |  | -4.42 | -4.2141 |
| mobley_6055410 |  | -9.4 | -12.7362 |
| mobley_6175884 |  | -4.27 | -3.9834 |
| mobley_6257907 |  | -5.82 | -6.6282 |
| mobley_6309289 |  | -5.11 | -3.9193 |
| mobley_632905 |  | -4.4 | -5.6389 |
| mobley_6416775 |  | 2.13 | 1.2506 |
| mobley_6474572 |  | -0.59 | 0.0169 |
| mobley_6620221 |  | -9.61 | -6.8768 |
| mobley_6632459 |  | -3.43 | -2.8627 |
| mobley_667278 |  | -4.4 | -3.1037 |
| mobley_6688723 |  | -6.44 | -6.8179 |
| mobley_6733657 |  | -3.44 | -0.8631 |
| mobley_6812653 |  | 2.48 | 0.9036 |
| mobley_6843802 |  | -1.29 | -1.299 |
| mobley_6861308 |  | -9.13 | -11.2232 |
| mobley_6935906 |  | -2.49 | -1.4086 |
| mobley_6978427 |  | -3.3 | -2.1294 |
| mobley_7176290 |  | -9.62 | -8.5872 |
| mobley_7203421 |  | -9.31 | -8.1794 |
| mobley_7227357 |  | -4.35 | -3.8512 |
| mobley_7261305 |  | -9.3 | -9.9765 |
| mobley_7375018 |  | -2.15 | -2.4192 |
| mobley_7415647 |  | -11.95 | -8.9219 |
| mobley_7497999 |  | -4.58 | -2.7106 |
| mobley_7542832 |  | -1.16 | -0.1404 |
| mobley_7573149 |  | -2.79 | -2.8432 |
| mobley_7599023 |  | -1.46 | -2.5388 |
| mobley_7608435 |  | -2.04 | -2.2934 |
| mobley_766666 |  | -0.44 | -1.6498 |
| mobley_7676709 |  | -4.51 | -2.4954 |
| mobley_7708038 |  | -0.56 | -0.3935 |
| mobley_7735340 |  | -9.63 | -5.8728 |
| mobley_7754849 |  | -6.1 | -7.2214 |
| mobley_7829570 |  | -3.51 | -4.07 |
| mobley_7860938 |  | -3.24 | -2.9753 |
| mobley_7893124 |  | -0.44 | -0.5195 |
| mobley_7913234 |  | -5.73 | -4.7875 |
| mobley_7943327 |  | -2.68 | -2.8244 |
| mobley_8057732 |  | -6.62 | -6.7847 |
| mobley_8127829 |  | -0.79 | -1.3819 |
| mobley_819018 |  | -4.45 | -4.458 |
| mobley_8311303 |  | -4.38 | -3.049 |
| mobley_8320545 |  | -3.71 | -1.8357 |
| mobley_8426916 |  | -4.07 | -3.8903 |
| mobley_8436428 |  | 2.83 | 1.6107 |
| mobley_8467917 |  | 1.68 | 0.2447 |
| mobley_8522124 |  | 0.71 | 0.2641 |
| mobley_8691603 |  | -5.49 | -6.2918 |
| mobley_8789864 |  | -6.4 | -5.9536 |
| mobley_8827942 |  | -4.05 | -4.4315 |
| mobley_8882696 |  | -3.47 | -0.7037 |
| mobley_8916409 |  | -8.15 | -8.5827 |
| mobley_900088 |  | 0.4 | 1.4838 |
| mobley_9007496 |  | -9.45 | -8.9672 |
| mobley_9055303 |  | 2 | 0.0169 |
| mobley_9100956 |  | 1.7 | 1.1343 |
| mobley_9121449 |  | -0.16 | -0.2743 |
| mobley_9197172 |  | 1.92 | 1.2814 |
| mobley_9209581 |  | -3.2 | -4.673 |
| mobley_9246351 |  | -1.96 | -1.6496 |
| mobley_9281946 |  | -6.74 | -6.5205 |
| mobley_929676 |  | 0.18 | 8.04E-03 |
| mobley_9507933 |  | -4.72 | -3.216 |
| mobley_9534740 |  | -25.47 | -23.6935 |
| mobley_9565165 |  | -3.35 | -2.3438 |
| mobley_9617923 |  | -2.34 | -2.5443 |
| mobley_9653690 |  | -4.44 | -3.2073 |
| mobley_9705941 |  | -1.38 | -1.0457 |
| mobley_9741965 |  | -4.29 | -5.4887 |
| mobley_9883303 |  | 2.71 | 1.3439 |
| mobley_9979854 |  | -4.2 | 1.1531 |

**C++ Source codes for the program to calculate hydration free energies**

/**

* Solvation Parameter Optimization Program.

* This program was written for optimization of solvation parameters used for docking simulation and MD simulation.

* If you want to obtain information about usage of solvation parameter and details,

* refer to Kang H., Choi H., Park H., J Chem Inf Model. 2007 Mar-Apr;47(2):509-14

*/

#include <iostream>

#include <vector>

#include <ga/GASimpleGA.h>

#include <ga/GABin2DecGenome.h>

#include <gsl/gsl_vector.h>

#include <gsl/gsl_multiroots.h>

#include <boost/tokenizer.hpp>

#include "mol2.h"

#include "cfg.h"

#include <string.h>

using namespace std;

extern FILE* yyin;

extern int yyparse(void);

extern int yylex(void);

string env_mode = "gaussian";

double sigma_sq = 3.50*3.50;

//#define env(ri, rj) exp(-dist_sq((ri), (rj))/(2*sigma_sq))

//#define env(ri, rj) exp(-dist_sq((ri), (rj))/(2*sigma_sq))/dist_sq((ri), (rj))

double (*env) (double ri[], double rj[]);

int selfterm = 0;

double abs_int(double a)

{

return (a > 0) ? a : -a;

}

float Calculate_Delta_G_Fixed_Volume(GAGenome& g);

float Calculate_Delta_G_Fixed_MaxOcc(GAGenome& g);

float Calculate_Delta_G_Fixed_MaxOcc_Fixed_Volume(GAGenome& g);

float Objective_Fixed_Volume(GAGenome& g);

float Objective_Fixed_MaxOcc(GAGenome& g);

float Objective_Fixed_MaxOcc_Fixed_Volume(GAGenome& g);

void usage();

void read_cfg(const char* cfgfile);

void toupper(string& s)

{

for(int i=0;i<s.length();i++)

{

s[i] = toupper(s[i]);

}

}

void get_range(string& value, double& range1, double& range2);

inline double dist_sq(double a[], double b[])

{

return ( (a[0]-b[0])*(a[0]-b[0]) + (a[1]-b[1])*(a[1]-b[1]) + (a[2]-b[2])*(a[2]-b[2]) );

}

double env_gaussian(double ri[], double rj[])

{

return exp(-dist_sq((ri), (rj))/(2*sigma_sq));

}

double env_scp(double ri[], double rj[])

{

return exp(-dist_sq((ri), (rj))/(2*sigma_sq))/dist_sq(ri, rj);

}

vector<Cfg_Atom> configurations;

typedef boost::tokenizer< boost::char_separator<char> > tokenizer;

boost::char_separator<char> sep("="), sep_range(":"), whitespace(" \t");

/**

* Global Variables

*/

vector<Mol2> molecules;

int num_of_trials = 10000;

double g_dCutOff = 0.01;

/**

* @structure rparams

* Structure for parameters.

* This structure contains two parameters used in multiple root finding method.

*/

struct rparams

{

double a;

double b;

};

int

rosenbrock_f (const gsl_vector * x, void *params,

gsl_vector * f)

{

double a = ((struct rparams *) params)->a;

double b = ((struct rparams *) params)->b;

const double x0 = gsl_vector_get (x, 0);

const double x1 = gsl_vector_get (x, 1);

const double y0 = a * (1 - x0);

const double y1 = b * (x1 - x0 * x0);

gsl_vector_set (f, 0, y0);

gsl_vector_set (f, 1, y1);

return GSL_SUCCESS;

}

GABoolean TerminateAtCutOff(GAGeneticAlgorithm & ga)

{

GABin2DecGenome & genome = (GABin2DecGenome &)(ga.statistics().bestIndividual());

double bestscore = ga.statistics().bestIndividual().score();

if(bestscore > g_dCutOff)

{

return gaTrue;

}

else

{

return gaFalse;

}

}

/**

* Main function.

*/

int main(int argc, char* argv[])

{

int popsize = 100;

int ngen = 1000;

int bits_per_gene = 6;

float pmut = 0.01;

float pcross = 0.6;

env = env_gaussian;

// const gsl_multiroot_fsolver_type *T;

// gsl_multiroot_fsolver *s;

// const size_t n = 2;

// struct rparams p = {1.0, 10.0};

// gsl_multiroot_function f = {&rosenbrock_f, n, &p};

// T = gsl_multiroot_fsolver_hybrids;

// s = gsl_multiroot_fsolver_alloc (T, 2);

// gsl_multiroot_fsolver_free (s);

opterr = 0;

int c;

string listfile, cfgfile, outfile, typefile, volumefile, line, ga_mode;

cfgfile = "default.cfg";

outfile = "bog.dat";

while((c = getopt(argc, argv, "l:c:n:p:m:s:o:t:v:d:e:f")) != -1)

{

switch(c)

{

case 'l':

listfile = optarg;

break;

case 'c':

cfgfile = optarg;

break;

case 'n':

ngen = atoi(optarg);

break;

case 'p':

popsize = atoi(optarg);

break;

case 'm':

pmut = atof(optarg);

break;

case 's':

pcross = atof(optarg);

break;

case 'o':

outfile = optarg;

break;

case 't':

typefile = optarg;

break;

case 'v':

volumefile = optarg;

break;

case 'd':

ga_mode = optarg;

break;

case 'e':

env_mode = optarg;

break;

case 'f':

selfterm = 1;

break;

default:

usage();

exit(1);

break;

}

}

if(env_mode == "scp")

{

env = env_scp;

}

if(listfile == "" || (typefile != "" && volumefile == "") || (typefile == "" && volumefile != ""))

{

usage();

exit(1);

}

cout << listfile << endl;

fstream mol2_f;

// Read mol2 files

mol2_f.open(listfile.c_str(), ios::in);

while(getline(mol2_f, line))

{

Mol2 m;

Mol2Reader reader(&m);

if(line.length() > 0 && line[0] != '#')

{

reader.read(line.c_str());

molecules.push_back(m);

}

}

mol2_f.close();

// Read configuration

#ifdef USE_LEX_YACC

while(!feof(stdin))

{

yyparse();

}

#else

read_cfg(cfgfile.c_str());

#endif

// Read volume type file

fstream voltype_f;

voltype_f.open(typefile.c_str(), ios::in);

string mode = "BEGIN", vt_mol2file;

for(int line_no=1;getline(voltype_f, line);line_no++)

{

if(line == "")

{

mode = "BEGIN";

}

else if(line.find("Volume Type") >= 0 && line.find("Volume Type") < line.length())

{

boost::char_separator<char> parenthesis("()");

tokenizer tokens(line, parenthesis);

tokenizer::iterator itr = tokens.begin();

if(itr == tokens.end()) { cerr << "Reading error (label) in volume type file : " << typefile << " Line #" << line_no << endl; return 1; }

itr++;

if(itr == tokens.end()) { cerr << "Reading error (label) in volume type file : " << typefile << " Line #" << line_no << endl; return 1; }

vt_mol2file = *itr;

mode = "ATOM";

}

else if(mode == "ATOM")

{

int atom_no, vol_type;

string atom_name;

tokenizer tokens(line, whitespace);

tokenizer::iterator itr = tokens.begin();

if(itr == tokens.end()) { cerr << "Reading error (first type) in volume type file : " << typefile << " Line #" << line_no << endl; return 1; }

atom_no = atoi(itr->c_str()); itr++;

if(atom_no <= 0) { cerr << "Reading error (second type) in volume type file : " << typefile << " Line #" << line_no << ". Atom number must be a positive integer" << endl; return 1; }

if(itr == tokens.end()) { cerr << "Reading error (third type) in volume type file : " << typefile << " Line #" << line_no << endl; return 1; }

atom_name = *itr; itr++;

if(itr == tokens.end()) { cerr << "Reading error (fourth type) in volume type file : " << typefile << " Line #" << line_no << endl; return 1; }

vol_type = atoi(itr->c_str()); itr++;

bool find_flag = false;

for(int mi=0;mi<molecules.size();mi++)

{

if(molecules[mi].filename == vt_mol2file)

{

molecules[mi].atoms[atom_no-1].atom_voltype = vol_type;

find_flag = true;

break;

}

}

// if(!find_flag) { cerr << "Reading error in volume type file : " << typefile << " Line #" << line_no << ". There is no atom matched with filename " << vt_mol2file << " atom number # " << atom_no << endl; return 1; }

}

}

voltype_f.close();

// Read volume file

fstream volume_f;

volume_f.open(volumefile.c_str(), ios::in);

map<int, double> volumes;

for(int line_no=1;getline(volume_f, line);line_no++)

{

int voltype_no;

double volume;

tokenizer tokens(line, whitespace);

tokenizer::iterator itr = tokens.begin();

if(itr == tokens.end()) { cerr << "Reading error in volume file : " << volumefile << " Line #" << line_no << endl; return 1; }

voltype_no = atoi(itr->c_str()); itr++;

if(itr == tokens.end()) { cerr << "Reading error in volume file : " << volumefile << " Line #" << line_no << endl; return 1; }

volume = atoi(itr->c_str()); itr++;

volumes[voltype_no] = volume;

}

volume_f.close();

for(int mi=0;mi<molecules.size();mi++)

{

for(int ai=0;ai<molecules[mi].atoms.size();ai++)

{

if(volumes.find(molecules[mi].atoms[ai].atom_voltype) != volumes.end())

{

molecules[mi].atoms[ai].volume = volumes[molecules[mi].atoms[ai].atom_voltype];

}

else

{

cerr << "Volume value was not found in file : " << volumefile << " type : " << molecules[mi].atoms[ai].atom_voltype << " molecule : " << molecules[mi].filename << " atom_no : " << ai << endl;

return 1;

}

}

}

unsigned int seed = 0;

GARandomSeed(seed);

GABin2DecPhenotype map;

for(int i=0;i<configurations.size();i++)

{

map.add(bits_per_gene, configurations[i].solpar_range[0], configurations[i].solpar_range[1]);

}

if((typefile == "" && volumefile == ""))

{

for(int i=0;i<configurations.size();i++)

{

map.add(bits_per_gene, configurations[i].volume_range[0], configurations[i].volume_range[1]);

}

}

// if(ga_mode == "volume" || strlen(volumefile.c_str()) > 0)

if(ga_mode == "volume")

{

for(int i=0;i<configurations.size();i++)

{

map.add(bits_per_gene, configurations[i].maxocc_range[0], configurations[i].maxocc_range[1]);

}

}

if(selfterm)

{

for(int i=0;i<configurations.size();i++)

{

map.add(bits_per_gene, configurations[i].selfterm_range[0], configurations[i].selfterm_range[1]);

}

}

string scorefile = outfile;

scorefile += ".bog";

GABin2DecGenome genome(map, (volumefile == "") ? Objective_Fixed_MaxOcc : ((ga_mode != "volume") ? Objective_Fixed_MaxOcc_Fixed_Volume : Objective_Fixed_Volume), NULL);

GASimpleGA ga(genome);

ga.populationSize(popsize);

ga.nGenerations(ngen);

ga.pMutation(pmut);

ga.pCrossover(pcross);

ga.flushFrequency(50); // dump scores to disk every 50th generation

ga.scoreFilename(scorefile.c_str());

// ga.terminator(TerminateAtCutOff);

for(int mi=0;mi<molecules.size();mi++)

{

for(int ai=0;ai<molecules[mi].atoms.size();ai++)

{

for(int ti=0;ti<configurations.size();ti++)

{

if(molecules[mi].atoms[ai].atom_type == configurations[ti].type)

{

molecules[mi].atoms[ai].atom_type_no = ti;

molecules[mi].atoms[ai].vol_type_no = ti+configurations.size();

}

}

if(molecules[mi].atoms[ai].atom_type_no > configurations.size())

{

cout << molecules[mi].atoms[ai].atom_type << endl;

}

}

}

genome.initialize();

ga.evolve(seed);

genome = ga.statistics().bestIndividual();

if(typefile == "")

{

Calculate_Delta_G_Fixed_MaxOcc(genome);

}

else if(ga_mode == "volume")

{

Calculate_Delta_G_Fixed_Volume(genome);

}

else

{

Calculate_Delta_G_Fixed_MaxOcc_Fixed_Volume(genome);

}

string resultcalcfile = outfile;

resultcalcfile += ".dg";

fstream resultcalc_f;

resultcalc_f.open(resultcalcfile.c_str(), ios::out);

for(int mi=0;mi<molecules.size();mi++)

{

resultcalc_f << molecules[mi].name << "\t" << molecules[mi].delta_g << "\t" << molecules[mi].delta_g_calc << endl;

}

resultcalc_f.close();

string solparfile = outfile;

solparfile += ".par";

fstream solpar_f;

solpar_f.open(solparfile.c_str(), ios::out);

for(int ci=0;ci<configurations.size();ci++)

{

solpar_f << configurations[ci].type << "\t" << genome.phenotype(ci) << endl;

}

solpar_f.close();

if(selfterm)

{

string selftermfile = outfile;

selftermfile += ".self";

fstream selfterm_f;

selfterm_f.open(selftermfile.c_str(), ios::out);

int st = configurations.size();

if((typefile == "" && volumefile == "")) st += configurations.size();

if(ga_mode == "volume") st += configurations.size();

for(int ci=0;ci<configurations.size();ci++)

{

selfterm_f << configurations[ci].type << "\t" << genome.phenotype(ci+st) << endl;

}

selfterm_f.close();

}

if(typefile == "")

{

string voloutfile = outfile;

voloutfile += ".vol";

fstream volout_f;

volout_f.open(voloutfile.c_str(), ios::out);

for(int ci=0;ci<configurations.size();ci++)

{

volout_f << configurations[ci].type << "\t" << genome.phenotype(ci+configurations.size()) << endl;

}

volout_f.close();

}

if(ga_mode == "volume")

{

string maxoccfile = outfile;

maxoccfile += ".occ";

fstream maxocc_f;

maxocc_f.open(maxoccfile.c_str(), ios::out);

for(int ci=0;ci<configurations.size();ci++)

{

maxocc_f << configurations[ci].type << "\t" << genome.phenotype(ci+configurations.size()) << endl;

}

maxocc_f.close();

}

return 0;

}

/**

* Usage. This function prints usage of this program.

*/

void usage()

{

cerr << "Usage: solpar -l [List of mol2] -c [Configuration File] -m [Mutaion Probability] -s [Cross Probability] -n [Number of generation] -p [Population Size] -t [Volume Type File] -v [Volume File] -o [Output File] -d [Mode] -e [Envelop Func] -f {Self-Solvation on}" << endl;

}

float Calculate_Delta_G_Fixed_Volume(GAGenome& g)

{

GABin2DecGenome & genome = (GABin2DecGenome &)g;

for(int mi=0;mi<molecules.size();mi++)

{

double dg_calc = 0.0;

for(int ai=0;ai<molecules[mi].atoms.size();ai++)

{

double occ = 0.0;

// assert(molecules[mi].atoms[ai].atom_type_no < 29);

if(molecules[mi].atoms[ai].atom_type_no >= configurations.size() || molecules[mi].atoms[ai].atom_type_no < 0)

{

cerr << molecules[mi].atoms[ai].atom_type_no << endl;

exit(1);

}

double si = genome.phenotype(molecules[mi].atoms[ai].atom_type_no);

for(int aj=0;aj<molecules[mi].atoms.size();aj++)

{

if(ai != aj)

{

double vj = molecules[mi].atoms[aj].volume;

occ += vj*env(molecules[mi].atoms[ai].pos, molecules[mi].atoms[aj].pos);

}

}

dg_calc += si*(genome.phenotype(molecules[mi].atoms[ai].atom_type_no + configurations.size()) - occ);

}

molecules[mi].delta_g_calc = dg_calc;

}

}

float Calculate_Delta_G_Fixed_MaxOcc(GAGenome& g)

{

GABin2DecGenome & genome = (GABin2DecGenome &)g;

static double sigma_sq = 3.50*3.50;

for(int mi=0;mi<molecules.size();mi++)

{

double dg_calc = 0.0;

for(int ai=0;ai<molecules[mi].atoms.size();ai++)

{

double occ = 0.0;

// assert(molecules[mi].atoms[ai].atom_type_no < 29);

if(molecules[mi].atoms[ai].atom_type_no >= configurations.size() || molecules[mi].atoms[ai].atom_type_no < 0)

{

cerr << molecules[mi].atoms[ai].atom_type_no << endl;

exit(1);

}

double si = genome.phenotype(molecules[mi].atoms[ai].atom_type_no);

for(int aj=0;aj<molecules[mi].atoms.size();aj++)

{

double vj = genome.phenotype(molecules[mi].atoms[aj].vol_type_no);

if(ai != aj)

{

occ += vj*env(molecules[mi].atoms[ai].pos, molecules[mi].atoms[aj].pos);

}

if(selfterm)

{

double sj = genome.phenotype(molecules[mi].atoms[aj].atom_type_no + configurations.size());

dg_calc += sj*vj*env(molecules[mi].atoms[ai].pos, molecules[mi].atoms[aj].pos);

}

}

dg_calc += si*(400.0 - occ);

}

molecules[mi].delta_g_calc = dg_calc;

}

}

float Calculate_Delta_G_Fixed_MaxOcc_Fixed_Volume(GAGenome& g)

{

GABin2DecGenome & genome = (GABin2DecGenome &)g;

static double sigma_sq = 3.50*3.50;

for(int mi=0;mi<molecules.size();mi++)

{

double dg_calc = 0.0;

for(int ai=0;ai<molecules[mi].atoms.size();ai++)

{

double occ = 0.0;

// assert(molecules[mi].atoms[ai].atom_type_no < 29);

if(molecules[mi].atoms[ai].atom_type_no >= configurations.size() || molecules[mi].atoms[ai].atom_type_no < 0)

{

cerr << molecules[mi].atoms[ai].atom_type_no << endl;

exit(1);

}

double si = genome.phenotype(molecules[mi].atoms[ai].atom_type_no);

for(int aj=0;aj<molecules[mi].atoms.size();aj++)

{

double vj = molecules[mi].atoms[aj].volume;

if(ai != aj)

{

occ += vj*env(molecules[mi].atoms[ai].pos, molecules[mi].atoms[aj].pos);

}

if(selfterm)

{

double sj = genome.phenotype(molecules[mi].atoms[aj].atom_type_no + configurations.size());

dg_calc += sj*vj*env(molecules[mi].atoms[ai].pos, molecules[mi].atoms[aj].pos);

}

}

dg_calc += si*(400.0 - occ);

}

molecules[mi].delta_g_calc = dg_calc;

}

}

float Objective_Fixed_Volume(GAGenome& g)

{

GABin2DecGenome & genome = (GABin2DecGenome &)g;

double fitness = 0.0;

static double sigma_sq = 3.50*3.50;

Calculate_Delta_G_Fixed_Volume(g);

for(int mi=0;mi<molecules.size();mi++)

{

double dg_exp = molecules[mi].delta_g;

double dg_calc = molecules[mi].delta_g_calc;

fitness += abs_int(dg_exp - dg_calc);

}

return 1.0/fitness;

}

float Objective_Fixed_MaxOcc(GAGenome& g)

{

GABin2DecGenome & genome = (GABin2DecGenome &)g;

double fitness = 0.0;

static double sigma_sq = 3.50*3.50;

Calculate_Delta_G_Fixed_MaxOcc(g);

for(int mi=0;mi<molecules.size();mi++)

{

double dg_exp = molecules[mi].delta_g;

double dg_calc = molecules[mi].delta_g_calc;

fitness += abs_int(dg_exp - dg_calc);

}

return 1.0/fitness;

}

float Objective_Fixed_MaxOcc_Fixed_Volume(GAGenome& g)

{

GABin2DecGenome & genome = (GABin2DecGenome &)g;

double fitness = 0.0;

static double sigma_sq = 3.50*3.50;

Calculate_Delta_G_Fixed_MaxOcc_Fixed_Volume(g);

for(int mi=0;mi<molecules.size();mi++)

{

double dg_exp = molecules[mi].delta_g;

double dg_calc = molecules[mi].delta_g_calc;

fitness += abs_int(dg_exp - dg_calc);

}

return 1.0/fitness;

}

void read_cfg(const char* cfgfile)

{

typedef boost::tokenizer< boost::char_separator<char> > tokenizer;

boost::char_separator<char> sep("="), sep_range(":");

fstream f;

string mode = "init";

f.open(cfgfile, ios::in);

char buf[1024] = {0,};

Cfg_Atom cfg_atm;

while(f.getline(buf, 1024))

{

string line = buf;

string upper_line = line;

toupper(upper_line);

if(line.find("//") >= 0 && line.find("//") < line.length())

{

line = line.substr(0, line.find("//"));

}

if(line.length() == 0)

{

if(mode == "atom")

{

configurations.push_back(cfg_atm);

cfg_atm.type = "";

cfg_atm.solpar_range[0] = cfg_atm.solpar_range[1] = cfg_atm.maxocc_range[0] = cfg_atm.maxocc_range[1] = cfg_atm.volume_range[0] = cfg_atm.volume_range[1] = cfg_atm.selfterm_range[0] = cfg_atm.selfterm_range[1] = 0.0;

}

mode == "init";

}

else if(mode == "atom")

{

tokenizer tokens(line, sep);

tokenizer::iterator itr = tokens.begin();

string key, value;

if(itr != tokens.end())

{

key = *itr;

itr++;

}

if(itr != tokens.end())

{

value = *itr;

itr++;

}

toupper(key);

if(key == "TYPE") { cfg_atm.type = value; }

if(key == "SOLPAR-RANGE")

{

get_range(value, cfg_atm.solpar_range[0], cfg_atm.solpar_range[1]);

}

if(key == "VOLUME-RANGE")

{

get_range(value, cfg_atm.volume_range[0], cfg_atm.volume_range[1]);

}

if(key == "MAXOCC-RANGE")

{

get_range(value, cfg_atm.maxocc_range[0], cfg_atm.maxocc_range[1]);

}

if(key == "SELFTERM-RANGE")

{

get_range(value, cfg_atm.selfterm_range[0], cfg_atm.selfterm_range[1]);

}

}

else if(mode == "init" && upper_line.find("[ATOM]") >= 0 && upper_line.find("[ATOM]") < upper_line.length())

{

mode = "atom";

}

}

configurations.push_back(cfg_atm);

f.close();

}

void get_range(string& value, double& range1, double& range2)

{

tokenizer value_tokens(value, sep_range);

tokenizer::iterator value_itr = value_tokens.begin();

if(value_itr != value_tokens.end())

{

range1 = atof(value_itr->c_str());

value_itr++;

}

if(value_itr != value_tokens.end())

{

range2 = atof(value_itr->c_str());

value_itr++;

}

}
